# RNA binding proteome profiling reveals RNA-dependent activation of oncogenic host signaling during KSHV infection

**DOI:** 10.1038/s41467-026-77861-x

**Published:** 2026-09-16

**Authors:** Yang Zhao, Xiang Ye, Madeline Yuen, Ruilin Zhang, W. Hayes McDonald, John Karijolich

**Affiliations:** 1 Department of Pathology, Microbiology and Immunology, Vanderbilt University Medical Center, Nashville, TN USA; 2 Vanderbilt Center for Immunobiology, Nashville, TN USA; 3 Vanderbilt Institute for Infection, Immunology, and Inflammation, Nashville, TN USA; 4 Vanderbilt Ingram Cancer Center, Nashville, TN USA; 5 Department of Biochemistry, Vanderbilt University, Nashville, TN USA; 6 Mass Spectrometry Research Center, Vanderbilt University, Nashville, TN USA

**Keywords:** Virology, Virus-host interactions

## Abstract

Viruses encode RNA-binding proteins (RBPs) to manipulate host processes and promote infection. However, due to limited genomic space, many viral proteins are multifunctional and may engage RNA through non-canonical domains, making their identification difficult using traditional sequence-based approaches. Kaposi’s sarcoma-associated herpesvirus (KSHV) is the etiological agent of many AIDS-associated cancers. Here, leveraging an unbiased proteomic approach we identified all RBPs in KSHV-infected primary effusion lymphoma (PEL) cells, uncovering 7 previously unrecognized KSHV-encoded RBPs. Among these is viral Interferon Regulatory Factor 1 (vIRF1), which we found is both necessary and sufficient to activate oncogenic RAS/MAPK signaling through an RNA-dependent interaction with host G3BP proteins. Importantly, a KSHV vIRF1 mutant lacking the ability to bind RNA exhibits a B cell-specific replication defect, highlighting the essential role of RNA interactions in viral gene regulation and pathogenesis. These findings reveal a novel strategy by which KSHV hijacks RNA-mediated signaling to support its lifecycle.

## Abstract (web summary)

Researchers mapped the RNA-binding proteins of a cancer-causing herpesvirus (KSHV) and found that the viral protein vIRF1 uses RNA to switch on a host growth-signaling pathway the virus needs to replicate in B cells.

## Introduction

Viruses rely heavily on cellular machinery for their replication and have evolved sophisticated strategies to hijack host processes to optimize infection. Central to this regulation is RNA, which not only serves as the template for protein synthesis but also functions as a structural and regulatory scaffold in diverse cellular pathways^1–3^. In addition, double-stranded RNA (dsRNA) is a well-established pathogen-associated molecular pattern (PAMP) that potently activates innate immune signaling^4,5^. Given these critical roles, it is not surprising that viruses encode proteins that interact with RNA to manipulate gene expression, evade immune detection, and facilitate replication. However, due to constraints in viral genome size, many viral proteins are multifunctional, and therefore potentially engage RNA through unconventional or poorly conserved domains, making their identification challenging using traditional domain-based prediction methods. Unbiased proteomic approaches offer a promising solution by enabling the comprehensive identification of RBPs—both cellular and viral—within infected cells and can uncover previously unrecognized layers of virus–host interaction^6^. Despite this potential, such approaches have remained underutilized.

In the context of viral infection, to date, global RBP discovery platforms have been applied exclusively to RNA viruses, where they have uncovered critical host RBPs that regulate viral replication^7,8^. In contrast, DNA viruses, despite their reliance on extensive RNA processing and host RNA regulatory pathways, remain unexplored in this context. This is a significant gap, particularly for large DNA viruses like herpesviruses, which encode dozens of proteins expressed in tightly regulated transcriptional programs and are known to manipulate nearly every aspect of host RNA biology.

Kaposi’s sarcoma-associated herpesvirus (KSHV) is a globally important AIDS-associated pathogen and the causative agent of multiple malignancies, including Kaposi’s sarcoma (KS) and the B cell lymphoma primary effusion lymphoma (PEL)^9–11^. Like all herpesviruses, KSHV persists through a biphasic lifecycle, alternating between latent and lytic phases^12,13^. Reactivation from latency and successful execution of the lytic gene expression program require precise coordination of viral and host gene expression. To accomplish this, KSHV encodes an array of regulatory factors that rewire host transcriptional and post-transcriptional pathways, including viral microRNAs and proteins that govern RNA stability, nuclear export, and immune evasion. Among these, several viral proteins, including K8 (K-bZIP), ORF37 (SOX), and ORF57, have been experimentally shown to directly interact with RNA within cells^14–16^. SOX acts as a host shutoff factor by promoting global mRNA degradation, ORF57 enhances the nuclear stability and export of viral transcripts, and K8, a transcriptional regulator with bZIP-like motifs, has been implicated in the regulation of lytic gene expression and viral DNA replication^15,17–19^. Notably, these proteins are multifunctional, and their established roles in KSHV biology are not solely dependent on RNA binding. In addition, ORF10, ORF26, ORF52, ORF59, and LANA have been reported to bind RNA in vitro; however, their direct RNA-binding activity in vivo remains undetermined^20–23^. Together, these observations suggest that RNA-protein interactions are a widespread and underappreciated layer of regulation in the KSHV lifecycle, with the potential to fine-tune diverse aspects of viral replication, immune evasion, and oncogenesis.

Here, we systematically defined the RBPome of KSHV-infected PEL cells during lytic reactivation using XRNAX, an unbiased proteomic approach for global RBP discovery^24^. This analysis recovered previously known KSHV-encoded RBPs, validating the method’s sensitivity, and identified 14 viral RBPs, including seven not previously implicated in RNA binding. Among these, we focused on viral interferon regulatory factor 1 (vIRF1), a multifunctional protein known for suppressing host interferon responses^25–28^. Surprisingly, an RNA-binding-deficient vIRF1 mutant exhibited cell-type-specific defects in viral gene expression and replication independent of its canonical immune antagonism. Mechanistically, our data suggest that vIRF1 activates the Ras/MAPK signaling pathway, which is essential for lytic reactivation, via an RNA-dependent interaction with the host scaffolding protein G3BP. These findings reveal a noncanonical, RNA-dependent function of vIRF1 in modulating host signal transduction. More broadly, this study expands the catalog of KSHV-encoded RBPs and highlights RNA binding as a central mechanism by which KSHV reprograms host cell biology. The resulting RBPome provides a rich resource for future exploration of RNA-mediated viral regulation and pathogenesis.

## Results

### Defining the RBPome of KSHV-infected PEL cells using XRNAX

**Fig. 1:**
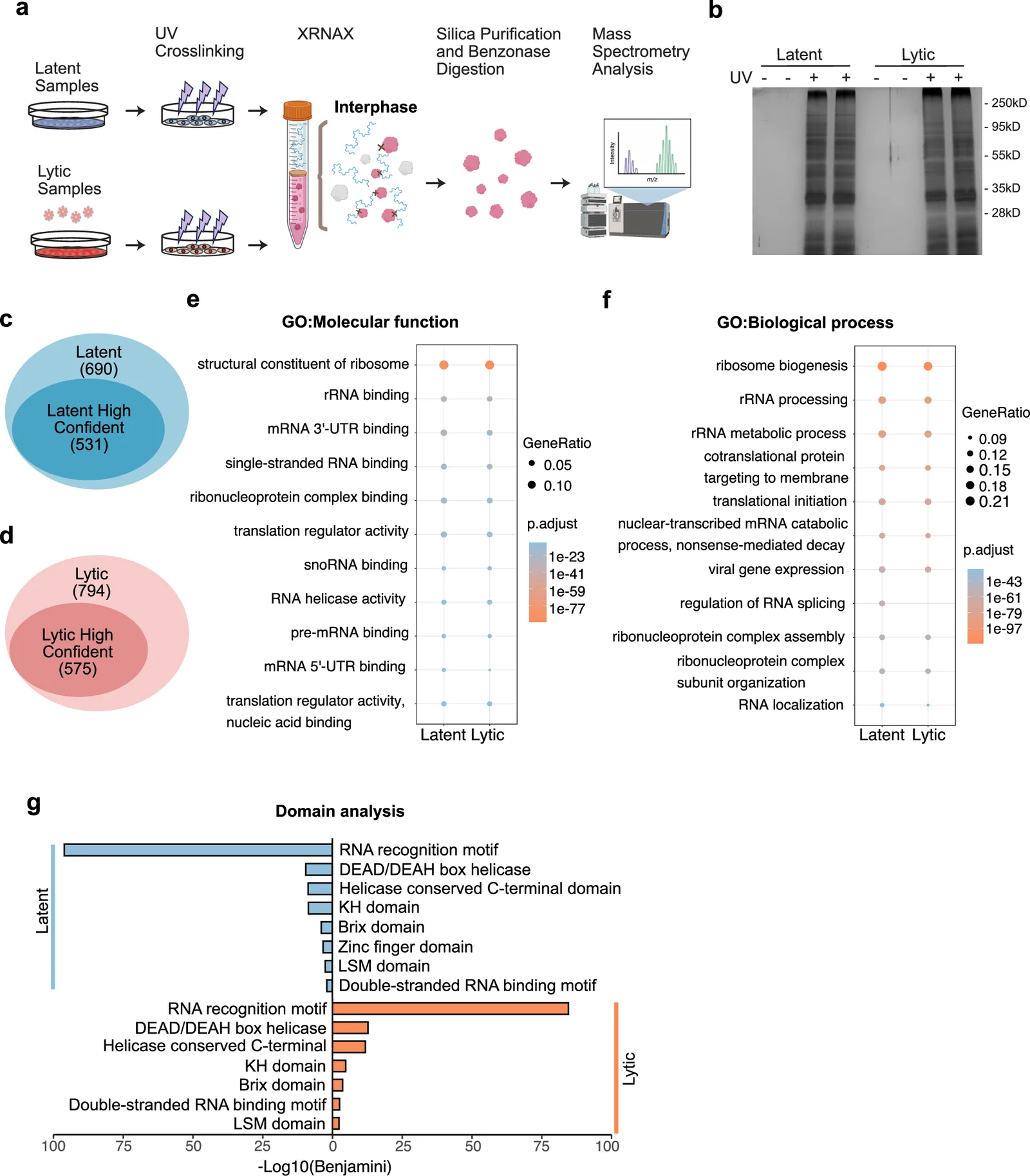
Systematic identification of RBPs in PEL cells with XRNAX. **a** Schematic of XRNAX assay of latent and lytic PEL cells. This image was created in BioRender (Created in Biorender. Karijolich, J. (2026) https://BioRender.com/iiyq3xf). **b** Representative SDS-PAGE and silver staining of proteins purified from UV 254 nm +/- latent and lytic TREx-BCBL1-RTA cells by XRNAX (*n* = 2). **c**,**d** Venn diagram showing the number of putative RBPs identified from latent and lytic TREx-BCBL1-RTA cells. High-confidence RBPs generate >2 unique peptides. **e**,**f** Molecular function (**e**) and biological processes (**f**) GO ontology of host RBPs identified in latent and lytic TREx-BCBL1-RTA cells. All human genes were used as background, and statistical significance was determined using Fisher’s exact test and the Benjamini-Hochberg method to correct for multiple testing. **g** RNA binding domain analysis of host RBPs identified in latent and lytic TREx-BCBL1-RTA cells. Source data are provided in Source Data file.

Given the central roles of RNA in gene regulation, innate immunity, and viral replication, we applied XRNAX to globally isolate RBPs from KSHV-infected PEL cells under both latent and lytic conditions (Fig. 1a). For these studies, we used TREx-BCBL1-RTA cells, a PEL-derived cell line that harbors a doxycycline (dox)-inducible transgene encoding the viral replication and transcription activator (RTA), allowing for controlled induction of the lytic cycle. Latently infected or dox-reactivated cells were UV-irradiated at 254 nm to covalently crosslink RNA–protein interactions in vivo. Crosslinked RNA–protein adducts were then enriched by acidic guanidinium thiocyanate–phenol–chloroform extraction, which traps the complexes at the interphase. Following recovery and resolubilization of the interphase material, RNA–protein complexes were further enriched using silica column purification, and subsequently eluted by RNase digestion to release the RNA-crosslinked proteins. To assess the efficiency and specificity of RBP recovery, a fraction of the eluate was resolved by SDS-PAGE and visualized by silver staining (Fig. 1b). Robust and diverse protein banding patterns were observed in eluates from UV-crosslinked samples, while little to no signal was detected in samples processed without UV treatment. These data confirm that protein recovery was dependent on UV crosslinking.

To comprehensively define the RBPome of KSHV-infected PEL cells, XRNAX eluates from both UV-crosslinked and non-crosslinked TREx-BCBL1-RTA cells were analyzed by high-performance liquid chromatography coupled to tandem mass spectrometry (HPLC-MS/MS). The number of RBPs in a given cell type can vary widely depending on cell lineage, physiological state, and environmental stimuli, and typically ranges from 300 to over 2000 proteins^1^. Qualitative analysis between crosslinked and non-crosslinked samples identified 690 and 794 candidate RBPs in latent and lytic reactivated cells, respectively (Fig. 1c, d and Supplementary Data 1, 2). The majority of candidate RBPs overlapped between the latent and lytic phases (Fig. S1a, b). Additionally, we identified 127 RBPs with significant differential abundance between UV-crosslinked latent and lytic samples (Supplementary data 3). However, further transcriptomic and proteomic analyses are required to determine whether these variations stem from changes in protein expression or RNA-binding. Gene ontology (GO) enrichment analysis confirmed that the proteins identified in our XRNAX preparations were highly enriched for RNA-related molecular functions and biological processes, including RNA binding, processing, and translation (Fig. 1e, f). Furthermore, domain analysis of the identified proteins revealed a strong enrichment for canonical RNA-binding domains, including RRM, KH, and DEAD-box motifs (Fig. 1g). These results validate the specificity and effectiveness of the XRNAX methodology in capturing RBPs and provide a robust dataset for investigating host and viral RNA–protein interactions during KSHV infection.

### Validation of KSHV RBPs

**Fig. 2:**
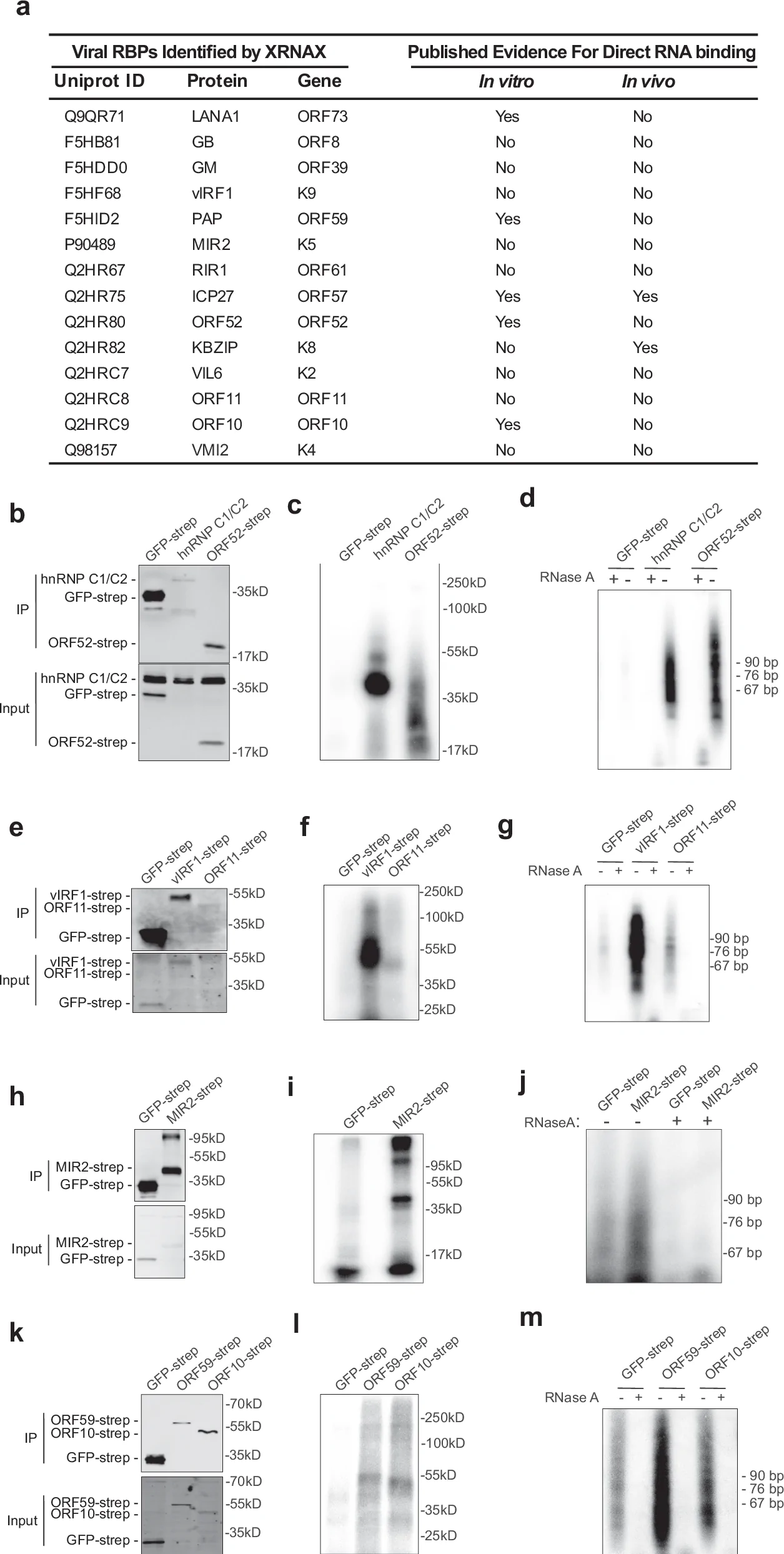
Validation of novel KSHV-encoded RBPs. **a** List of putative RBPs encoded by KSHV. **b**,**e**,**h**,**k** Constructs expressing strep-tagged viral proteins were transfected to TREx-BCBL1-RTA cells and CLIP was performed. Representative western blot analysis of input and IP for the indicated samples (*n* = 3). **c**,**f**,**i**,**l** Representative ^32^P-autoradiograph of the IP samples (*n* = 3). **d**,**g**,**j**,**m**, Representative urea-PAGE of nucleic acid that was immunoprecipitated from the indicated samples with RNase A -/+ treatment. Source data are provided in Source Data file.

To define the landscape of KSHV-encoded RBPs, we interrogated our mass spectrometry dataset for viral proteins enriched in the RBPome. Our XRNAX data identified 14 candidate viral RNA-binding proteins (RBPs) (Fig. 2a). Within these proteins, we identified ORF57 and K8 (K-bZIP), previously reported to bind RNA directly in vivo. We also confirmed direct RNA-binding in vivo for LANA, ORF52, ORF59, and ORF10, which have been reported to bind RNA in vitro or associate with RNA in cellular coimmunoprecipitation assays. Additionally, we discovered 7 candidate viral RBPs not previously reported: GB (ORF8), GM (ORF39), vIRF1 (K9), MIR2 (K5), RIR1 (ORF61), vIL6 (K2), and vMI2 (K4). These candidates span both latent and lytic gene classes and include proteins lacking canonical RNA-binding domains, suggesting that KSHV may utilize multifunctional or structurally unconventional domains to engage RNA. Intriguingly, we did not capture ORF37 and ORF26. As ORF37 functions as an endonuclease that promotes global mRNA degradation, the transient nature of the interaction between the nuclease and its substrate may make it challenging to capture using XRNAX. Regarding ORF26, we speculate that its reported interaction with RNA in the in vitro affinity purification assays might be mediated by other proteins within cells, as the XRNAX method is specifically designed to capture only direct RNA-protein interactions via covalent UV cross-linking.

To validate direct RNA binding, we performed UV crosslinking and immunoprecipitation (CLIP) assays coupled to ^32^P labeling. We selected six candidates, ORF52, vIRF1 (K9), ORF10, ORF11, MIR2 (K5), PAP (ORF59), for verification based on our ability to consistently detect their expression as well as immunoprecipitate them. We note that although ORF10 and ORF52 RNA binding was demonstrated in vitro, their ability to bind RNA directly within cells remained untested, and therefore they were included here. Strep-tagged versions of each protein, along with strep-GFP (negative control), were individually nucleofected into TREx-BCBL1-RTA cells, followed by lytic reactivation with dox for 48 h. Cells were UV-crosslinked at 254 nm, lysed, and strep-tagged proteins were immunoprecipitated under stringent conditions from RNase T1-digested extracts. To detect covalently bound nucleic acid, eluates were ^32^P-labeled using polynucleotide kinase (PNK), resolved by SDS-PAGE, and visualized by autoradiography. As expected, strep-GFP showed minimal signal, whereas endogenous hnRNP C1/C2 (positive control) yielded a strong ^32^P-labeled band. Strikingly, all the viral proteins tested displayed robust ^32^P signals, indicating direct crosslinking to nucleic acid in cells (Fig. 2b, c, e, f, h, i, k, l). To confirm the labeled nucleic acid was RNA, proteinase K–treated eluates were treated with RNase A and resolved by urea-PAGE. In all cases, the ^32^P signal was abolished by RNase A (Fig. 2d, g, j, m), validating their ability to directly bind RNA in cells. Collectively, these data confirm ORF52, vIRF1, ORF10, ORF11, MIR2, and PAP as KSHV-encoded RBPs and underscore the expanding role of RNA-binding proteins in viral biology.

### vIRF1 RNA-binding is critical for KSHV lytic reactivation in B cells

Among the newly identified KSHV RBPs, we focused on vIRF1, as it has homology with the human interferon regulatory factor (IRF) family and is expressed throughout the viral lifecycle^28^. vIRF1 is implicated in a wide range of cellular processes, including cell transformation and the inhibition of interferon responses^25–35^. Structurally, vIRF1 contains an N-terminal DNA-binding domain (DBD) and a C-terminal IRF association domain (IAD). Despite the DBD, it is generally considered that vIRF1 cannot bind DNA, as full-length vIRF1 does not bind DNA in vitro, nor is it able to bind consensus IRF DNA sequences in cells or in vitro^23,24,33^.

**Fig. 3:**
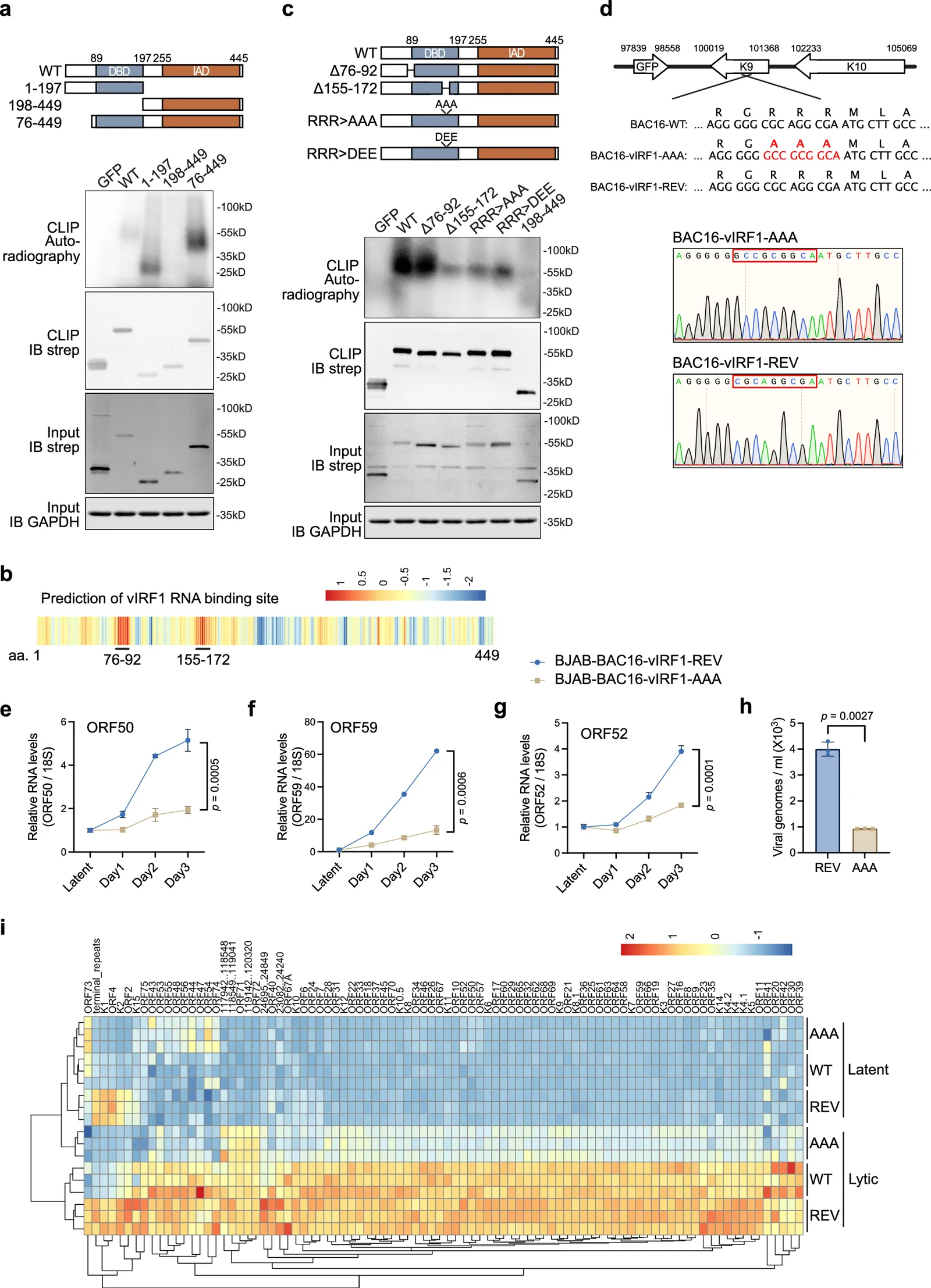
Identification of RNA binding motif in vIRF1 and characterization of its critical function for KSHV lytic reactivation in B cells. **a**,**c** Identification of vIRF1 RNA binding site by ^32^P-CLIP. Representative western blot analysis and ^32^P-autoradiograph of the indicated samples (*n* = 3). **b** Computational prediction of the vIRF1 RNA binding site. **d** Sanger sequencing to validate the BAC16 vIRF1 mutant AAA (RRR_161-163_ → AAA_161-163_) and the revertant REV (AAA_161-163_ → RRR_161-163_) **e–g** RT-qPCR analysis of indicated lytic genes expression in BJAB-BAC16 cells with AAA and REV vIRF1 viruses. Lytic reactivation was induced with 20 ng/ml TPA and 1 mM NaB. **h** RT-qPCR quantification of virion production in samples as in (**e**). **i** Heatmap of KSHV gene expression of latent and lytic BJAB-BAC16 cells with WT, AAA and REV vIRF1 viruses. RT-qPCR data are presented as mean values ± SD (*n* = 3 biologically independent samples). *p*-values were determined by the two-tailed Welch’s *t*test. Source data are provided in Source Data file.

To narrow the region responsible for RNA binding, we generated a series of strep-tagged vIRF1 deletion mutants targeting either the DBD, the IAD, or sequences flanking these domains, and expressed them in TREx-BCBL1-RTA cells (Fig. 3a). Following lytic reactivation, we performed UV CLIP assays coupled with ^32^P labeling. These experiments revealed that the RNA-binding activity maps to the N-terminal DBD region, whereas deletion of the C-terminal IAD had no effect (Fig. 3a).

To more precisely define the RNA-binding interface within vIRF1, we leveraged the structure-informed prediction tool pPrint^36^, which identified two candidate RNA-binding regions located within the DBD: amino acids 76–92 and 155–172 (Fig. 3b). To assess the functional contribution of each region, we generated internal deletion mutants lacking either sequence and tested their RNA-binding activity using UV CLIP coupled with ^32^P labeling. Deletion of amino acids 76–92 had no appreciable effect, whereas deletion of residues 155–172 resulted in a substantial loss of RNA binding, implicating this region as critical for interaction with RNA (Fig. 3c). Within the 155–172 segment, pPrint further predicted a cluster of three arginine amino acids (RRR _161-163_) as key RNA-contacting residues. To directly test this prediction, we mutated the arginine to alanine (vIRF1-AAA) or substituted them with negatively charged residues (vIRF1-DEE). Both mutations significantly reduced RNA binding in ^32^P-CLIP assays, confirming that this motif is critical for vIRF1’s ability to engage RNA (Fig. 3c).

To test whether vIRF1 RNA binding plays a role in the KSHV lifecycle, we generated a KSHV BAC16 mutant in which the three arginine residues involved in RNA binding were mutated to alanine (vIRF1-AAA). In addition, we constructed the wild-type revertant (vIRF1-REV). vIRF1-AAA and vIRF1-REV BAC16 were validated by Sanger sequencing and restriction enzyme digestion (Fig. 3d and S2a). KSHV vIRF1-AAA and vIRF1-REV infections were established in iSLK-RTA and BJAB cells. iSLK-RTA cells harbor a dox-inducible RTA that provides the opportunity to reactivate KSHV by the addition of dox to the culture media, and BJAB-BAC16 cells were reactivated with 12-O-tetradecanoylphorbol-13-acetate (TPA) and sodium butyrate (NaB). While iSLK cells are of epithelial cell origin and not of known physiological relevance, BJABs are B cells which are a natural latent reservoir for KSHV. Similar levels of genomes were present across all latent infections (Fig. S2b, c). In addition, western blot analysis of cytoplasmic and nuclear fractions revealed no observable localization (Fig. S2d, e). Following the addition of dox, we quantified viral gene expression in iSLK-RTA cells across a three-day time course by RT-qPCR of select viral genes and observed that AAA and revertant viruses reactivated to a similar extent (Figure S2f-h). However, in BJAB cells, BAC16 vIRF1-AAA exhibited a significant reduction in viral gene expression relative to the revertant (Fig. 3e–g). Total RNA sequencing of latent and lytically reactivated BJAB-BAC16 WT, vIRF1-AAA, and vIRF1-REV cells indicated that latent gene expression was comparable between all three conditions, with only minor variations observable. In contrast, lytic-reactivated BAC16 vIRF1-AAA had a general and profound defect in viral gene expression when compared to BAC16 WT and vIRF1-REV (Fig. 3i). Moreover, BAC16 vIRF1-AAA produced significantly less encapsidated viral DNA (Fig. 3h).

To rule out the possibility that the observed replication defect of the vIRF1-AAA mutant was specific to TPA/NaB-mediated reactivation, we further tested two independent approaches to induce KSHV lytic reactivation in B cells. First, we generated BJAB-iRTA cells by integrating a dox-inducible RTA transgene using Sleeping Beauty–mediated transposition. We then established latent infections with vIRF1-AAA and vIRF1-REV viruses and monitored lytic reactivation following dox treatment. Second, we tested this phenotype using B cell receptor (BCR) crosslinking with anti-IgM, a physiologically relevant stimulus that mimics B cell activation. In both systems, vIRF1-AAA showed consistently impaired lytic replication, suggesting that the RNA-binding activity of vIRF1 is essential for productive reactivation in B cells and that this defect is not restricted to a specific reactivation stimulus (Fig. S2i, j). To test whether this observation extends beyond BJABs and into other B cell models, we established latent infections with vIRF1-AAA and vIRF1-REV viruses in Epstein-Barr virus (EBV) negative Akata cells^37^, a Burkitt’s lymphoma cell line that has spontaneously lost its EBV genomes. Consistent with our observations in BJABs, vIRF1-AAA virus has a pronounced lytic gene expression defect when induced with TPA and NaB (Fig. S2k, l). This suggests that the lytic gene expression phenotype may be a general B cell-specific characteristic, rather than restricted to the BJAB cell line. Collectively, these data establish vIRF1 as an RBP and reveal its previously unrecognized RNA-binding-motif-dependent role in facilitating KSHV lytic replication within its B cell reservoir.

### vIRF1 RNA-dependent MAPK Activation Facilitates KSHV Reactivation

To determine the mechanism by which vIRF1 RNA-binding facilitates KSHV lytic reactivation, we first tested whether loss of RNA binding alters vIRF1’s ability to suppress the interferon (IFN) response, a function for which vIRF1 was originally characterized. We transfected HEK293T cells with constructs expressing GFP, wild-type (WT) vIRF1, or the RNA-binding-deficient vIRF1-AAA mutant, along with an ISRE-luciferase reporter and key activators of the nucleic acid sensing pathway (MAVS, TBK1, IKKε, and IRF3). Both WT and vIRF1-AAA robustly suppressed ISRE-driven luciferase activity across all four conditions, comparable to each other and significantly more than the GFP control (Fig. S3a–d). To extend these findings to an infection context, we also examined IFN and NF-κB signaling in BJAB-BAC16 cells with WT, vIRF1-AAA, or vIRF1-REV viruses, and again observed no significant differences in pathway activation among the three (Fig. S3e, f). These results indicate that the lytic reactivation defect observed in vIRF1-AAA–infected B cells is not attributable to altered interferon suppression, and point instead to a distinct, RNA-dependent function of vIRF1 in regulating KSHV replication.

**Fig. 4:**
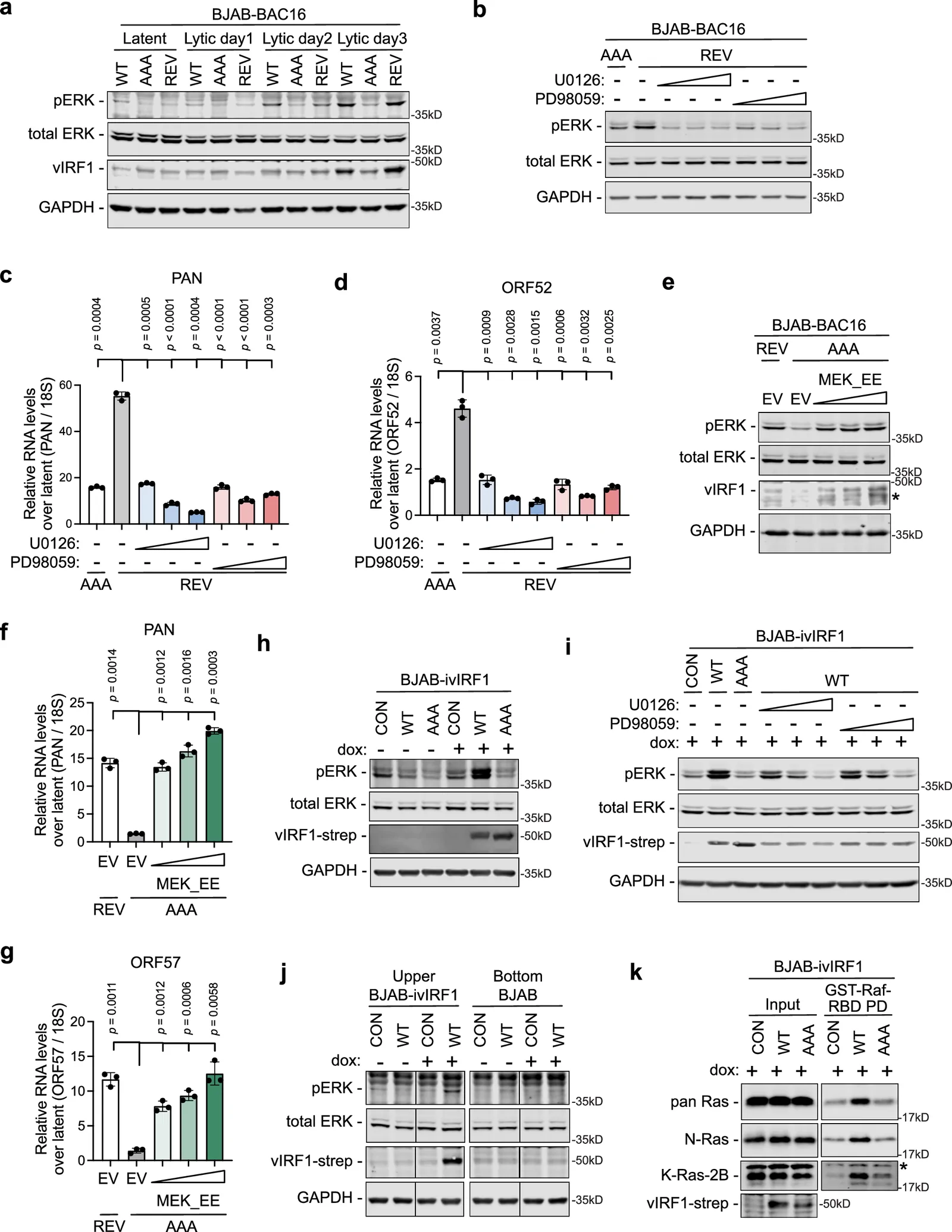
vIRF1 RNA-dependent ERK activation and its role in KSHV reactivation in B cells. **a** Representative western blot of protein extracts from BJAB-BAC16 cells reactivated for up to 3 days with TPA and NaB (*n* = 3). **b–d** Western blot (**b**) and RT-qPCR (**c,d**) of samples from BJAB-BAC16 vIRF1-REV cells treated with MEK inhibitors, U0126 and PD98059. **e–g**, Western blot (**e**) and RT-qPCR (**f**,**g**) of samples from BJAB-BAC16 vIRF1-AAA cells microporated with constructs expressing constitutively active MEK (MEK_EE). **h** Representative western blot of protein extracts from BJAB cells expressing dox-inducible, strep-tagged WT vIRF1 or the RNA-binding–defective mutant vIRF1-AAA (*n* = 3). **i** Representative western blot of protein extracts from BJAB cells expressing WT vIRF1 treated with MEK inhibitors, U0126 and PD98059 (*n* = 3). **j** Representative western blot of protein extracts from a transwell co-culture assay using BJAB cells expressing WT vIRF1 in the upper chamber and naïve BJAB cells in the lower chamber (*n* = 3). **k** Representative western blot of protein extracts from active Ras pull-down assay of BJAB cells expressing dox-inducible WT vIRF1 or mutant vIRF1-AAA (*n* = 3). * indicate non-specific bands. RT-qPCR data are presented as mean values ± SD (*n* = 3 biologically independent samples). *p*-values were determined by the two-tailed Welch’s *t*test. Source data are provided in Source Data file.

MAPK signaling is an established regulator of KSHV lytic reactivation, with ERK1/2 activation playing a critical role in initiating the viral gene expression cascade^38^. To assess whether impaired RNA binding by vIRF1 affects MAPK activation, we monitored phosphorylation of ERK1/2, p38, and JNK in BJAB-BAC16 cells harboring WT, vIRF1-AAA, or vIRF1-REV viruses. In cells infected with WT or vIRF1-REV virus, phospho-ERK1/2 levels steadily increased over a 3-day course of lytic reactivation, consistent with initiation of the lytic program (Fig. 4a). In contrast, vIRF1-AAA virus-infected BJAB cells exhibited markedly reduced phospho-ERK1/2, indicating a failure to activate this signaling axis. Phosphorylated p38 and JNK were undetectable across all conditions (Fig. S4a), suggesting ERK1/2 is the predominant MAPK pathway activated in this context. The defect in ERK1/2 activation in vIRF1-AAA cells was confirmed using two independent B cell reactivation models—IgM-mediated BCR crosslinking and doxycycline-inducible RTA in BJAB-iRTA cells (Fig. S4b, c), as well as in another B cell line, the EBV-negative Akata cells (Fig. S4d). Notably, this defect was absent in iSLK-RTA cells, where ERK1/2 phosphorylation levels were comparable between WT and vIRF1-AAA viruses (Fig. S4e). These results identify a cell type–restricted role for vIRF1 in promoting MAPK signaling, and strongly implicate RNA binding as essential for the activation of a host pathway critical for KSHV lytic replication.

The striking loss of phospho-ERK1/2 in vIRF1-AAA BAC16–infected B cells suggested that defective MAPK signaling may be the primary driver of the impaired lytic gene expression phenotype. To test this hypothesis, we first validated the requirement for ERK1/2 activation during KSHV lytic reactivation in BJAB-BAC16 cells. Treatment with two independent small-molecule MEK1/2 inhibitors, the kinase upstream of ERK1/2, efficiently suppressed ERK phosphorylation (Fig. 4b). Importantly, this was accompanied by a significant decrease in KSHV lytic gene expression, confirming the essential role of the MEK–ERK axis in promoting viral reactivation (Fig. 4c, d).

To further associate the lytic replication defect with a failure to activate MAPK signaling, we tested if constitutively active MEK would rescue BAC16 vIRF1-AAA viral gene expression. Indeed, nucleofection of a constitutively active MEK1 mutant (MEK_EE; S218E/S222E^39,40^) into vIRF1-AAA BAC16 cells robustly restored ERK1/2 phosphorylation and fully rescued viral lytic gene expression (Fig. 4e–g). These results provide direct evidence that failure to activate ERK1/2 underlies the reactivation defect in vIRF1-AAA cells, and establish that vIRF1-mediated MAPK activation is a critical RNA-binding–motif dependent mechanism by which KSHV drives its lytic replication program in B cells.

Having established that mutation of the vIRF1 RNA-binding motif disrupts MAPK signaling during KSHV infection in B cells we next tested whether vIRF1 is sufficient to drive ERK1/2 activation. To test this, we generated BJAB cells expressing dox-inducible, strep-tagged WT vIRF1 or the RNA-binding–defective mutant vIRF1-AAA (BJAB-ivIRF1 WT and AAA, respectively). Upon dox induction, western blot analysis revealed a strong increase in phospho-ERK1/2 levels exclusively in cells expressing WT vIRF1, but not in those expressing the vIRF1-AAA mutant, indicating that the RNA-binding motif is essential for this activity (Fig. 4h). Moreover, pharmacologic inhibition of MEK, the upstream kinase of ERK1/2, completely abrogated vIRF1-mediated ERK1/2 phosphorylation, confirming that vIRF1 acts at or upstream of MEK (Fig. 4i). Together, these results demonstrate that vIRF1 is not only necessary but also sufficient to activate MAPK signaling in B cells, and that this function is dependent on the RNA binding motif.

Activation of the MAPK signaling cascade can be initiated by soluble ligands, such as cytokines and growth factors, that engage membrane-bound receptor tyrosine kinases, leading to Ras activation and downstream ERK phosphorylation^41–43^. To determine whether vIRF1 promotes MAPK signaling through a secreted factor, we conducted a transwell co-culture assay using BJAB-ivIRF1 cells (expressing WT vIRF1) in the upper chamber and naïve BJAB cells in the lower chamber, separated by a 0.45 μm pore membrane. After 72 h of dox-induced vIRF1 expression, phospho-ERK1/2 levels were assessed in both compartments by western blot. As expected, ERK1/2 phosphorylation was robust in vIRF1-expressing cells in the upper chamber. However, no ERK1/2 activation was detected in the naïve BJAB cells below, indicating that vIRF1-mediated MAPK activation is not driven by a diffusible, soluble ligand but rather occurs via a cell-intrinsic mechanism (Fig. 4j).

Given the critical role of Ras upstream of MEK-ERK signaling, we next tested whether vIRF1 influences Ras activity. To this end, we performed a Ras-GTP pulldown assay using the Ras-binding domain (RBD) of the Raf1 kinase, which selectively binds the active, GTP-bound form of Ras with high affinity and stabilizes it by inhibiting GTP hydrolysis. Using this approach, we found that expression of WT vIRF1 significantly increased Ras-GTP levels relative to control BJAB cells, whereas the RNA-binding–deficient vIRF1-AAA mutant failed to promote Ras activation (Fig. 4k). These findings demonstrate that vIRF1 enhances MAPK signaling by promoting Ras activation through an RNA-binding-motif–dependent, cell-intrinsic mechanism.

### vIRF1 RNA-dependent protein interactions

**Fig. 5:**
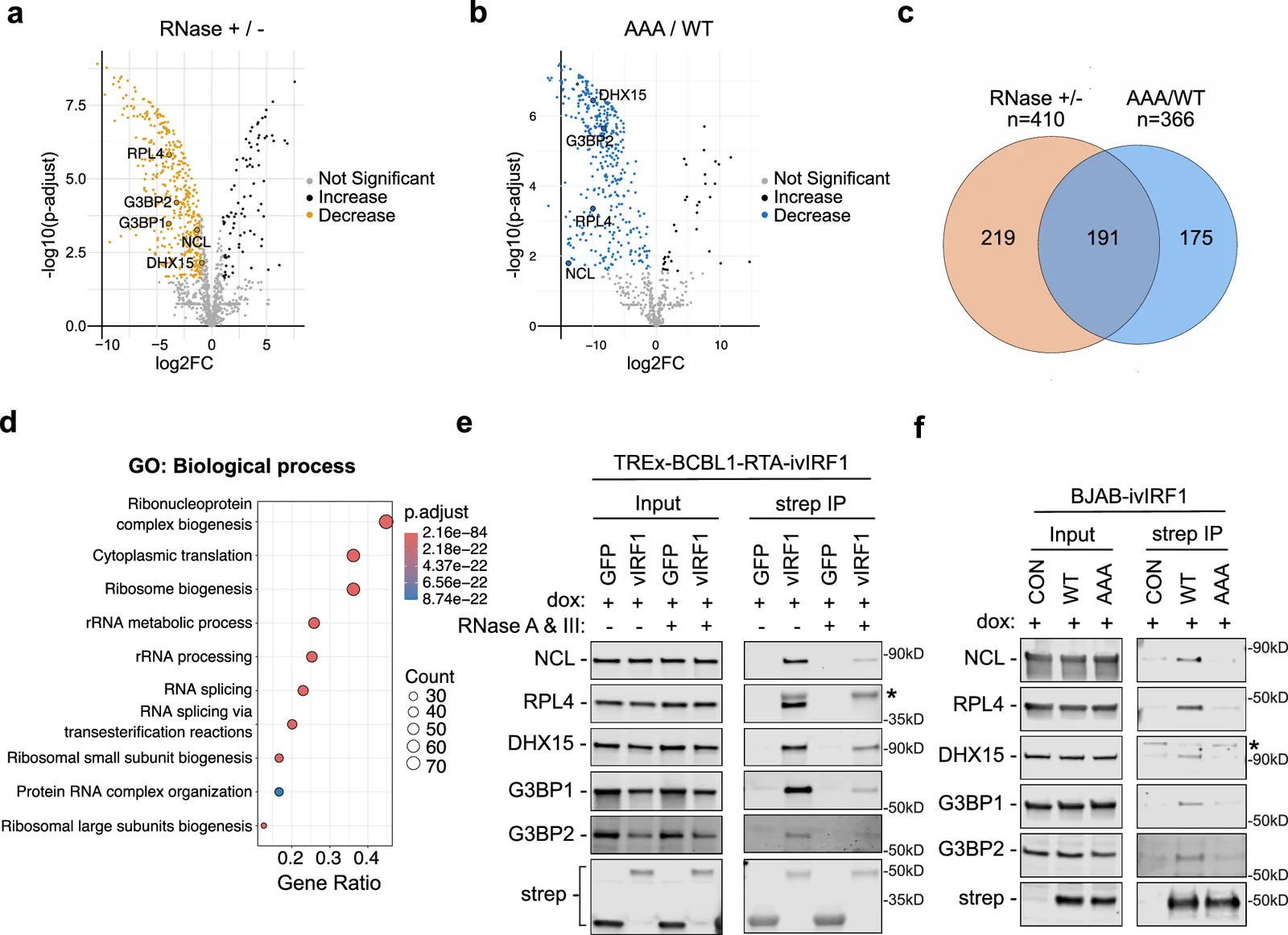
vIRF1 RNA-dependent protein interactions. **a** Volcano plot showing the log2 fold change and -log10 p.adjust of proteins identified from strep-tagged vIRF1 IP of TREx-BCBL1-RTA cells in the condition of RNase A/III treatment. The tan colored dots indicated the proteins that have a significant decrease of binding with vIRF1 with RNase treatment (log2FC < −1 and p.adjust<0.05). **b** Volcano plot showing the log2 fold change and -log10 p.adjust of proteins identified from strep-tagged WT and AAA vIRF1 IP of TREx-BCBL1-RTA cells. The blue colored dots indicated the proteins that have a significant decrease of binding with AAA vIRF1 comparing with WT (log2FC < −1 and p.adjust<0.05). The empirical Bayesian method moderated *t*test was applied to determine *p*-values and p values adjusted for multiple-testing were determined using Benjamini-Hochberg method in (**a**) and (**b**). **c** Venn diagram showing the overlap of the proteins having significant decreased binding in (**a**) and (**b**). **d** GO analysis of the overlapping proteins in (**c**). Statistical significance was determined using the Fisher’s exact test and Benjamini-Hochberg method to correct for multiple testing. **e** Representative western blot of representative proteins of strep-tagged vIRF1 IP of TREx-BCBL1-RTA cells in the condition of RNase A/III treatment (*n* = 3). **f** Representative western blot of representative proteins of strep tagged-vIRF1 WT and AAA IP of BJAB-ivIRF1 cells (*n* = 3). * indicate non-specific bands. Source data are provided in Source Data file.

Given that vIRF1-mediated activation of the Ras/MAPK pathway is both cell-intrinsic and dependent on the RNA-binding motif we hypothesized that vIRF1 promotes signaling by engaging host proteins through RNA-scaffolded interactions. To test this, we engineered TREx-BCBL1-RTA cells to express dox-inducible, strep-tagged GFP or WT vIRF1. We first performed affinity purification of WT vIRF1 in the presence or absence of RNase A/III to distinguish direct protein-protein interactions from those bridged by RNA. Mass spectrometry of the resulting immunoprecipitates identified 410 proteins whose interaction with vIRF1 was significantly reduced upon RNase treatment (Fig. 5a and Supplementary Data 4). To complement this approach, we nucleofected strep-tagged GFP, WT and AAA mutant vIRF1 into TREx-BCBL1-RTA cells and carried out parallel affinity purifications and compared their interactomes. This analysis revealed 366 proteins whose binding to vIRF1 was significantly diminished in the AAA mutant relative to WT (Fig. 5b and Supplementary Data 5). Intersecting the RNase-sensitive and mutation-sensitive datasets yielded a high-confidence set of 191 proteins that associate with vIRF1 in an RNA-dependent manner and require an intact RNA-binding interface (Fig. 5c).

Gene ontology enrichment analysis of the 191 proteins revealed striking overrepresentation of RNA-centric functions, including RNA processing, translation, and pre-mRNA splicing, highlighting the central role of host RNA-protein networks in vIRF1 function (Fig. 5d). To validate these findings, we selected five representative interactors and confirmed their RNA-dependent association with vIRF1 by co-immunoprecipitation and western blotting. These interactions were abolished by RNase treatment and selectively disrupted in the vIRF1 AAA mutant (Fig. 5e, f). While these interactions were recovered from extracts and the precise cellular context of their assembly remains to be defined, their abolition by RNase and selective loss in the vIRF1-AAA mutant together supports that vIRF1 engages this host RNA–protein network through its RNA-binding activity.

### An RNA-dependent vIRF1-G3BP interaction facilitates MAPK activation

Among the validated interactors, the G3BP proteins emerged as particularly compelling candidates. Although best known for their role in stress granule formation, they are also ontologically associated with Ras protein signal transduction and small GTPase-mediated signaling^44–46^. Given this dual role, we hypothesized that vIRF1 co-opts G3BPs through RNA-mediated scaffolding to promote Ras/MAPK pathway activation.

**Fig. 6:**
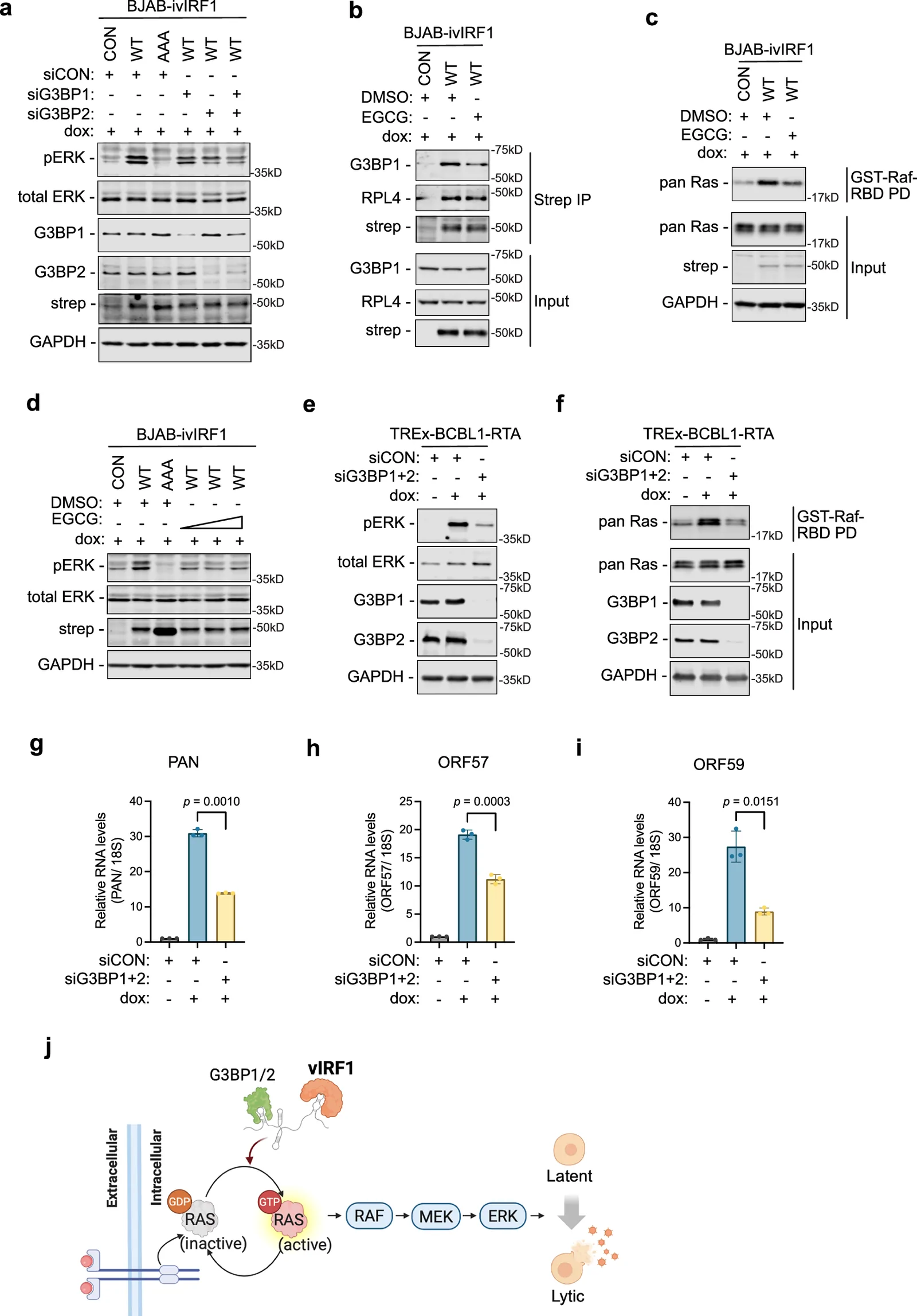
G3BP 1/2 are required for vIRF1-induced ERK activation. **a** Representative western blot of inducible vIRF1 BJAB cells knocking down G3BP proteins with siRNA (*n* = 3). **b** Representative western blot of strep-tagged vIRF1 IP in inducible vIRF1 expressing BJAB cells with EGCG treatment (*n* = 3). **c** Representative western blot of samples from active Ras pull down assay of inducible vIRF1 expressing BJAB cells with EGCG treatment (*n* = 3). **d** Representative western blot protein extracts from inducible vIRF1 expressing BJAB cells with EGCG treatment (*n* = 3). **e** Representative western blot of cell lysates from TREx-BCBL1-RTA cells knocking down G3BP proteins with siRNA (*n* = 3). **f** Representative western blot of samples from active Ras pull down assay of TREx-BCBL1-RTA cells knocking down G3BP proteins with siRNA (*n* = 3). **g–i** RT-qPCR of PAN (**g**), ORF57 (**h**) and ORF59 (**i**) expression in TREx-BCBL1-RTA cells knocking down G3BP proteins with siRNA. **j** The model depicting how vIRF1 promotes KSHV lytic reactivation in an RNA dependent manner. This image was created in Biorender (Created in Biorender. Karijolich, J. (2026) https://BioRender.com/eqxwjo1). RT-qPCR data are presented as mean values ± SD (*n* = 3 biologically independent samples). *p*-values were determined by the two-tailed Welch’s *t*test. Source data are provided in Source Data file.

To test whether G3BPs are functionally required for vIRF1-mediated activation of the MAPK pathway, we first examined the impact of G3BP1 and G3BP2 depletion on ERK phosphorylation. Using BJAB cells harboring dox-inducible WT vIRF1, we performed siRNA-mediated knockdown of G3BP1 or G3BP2 and assessed ERK activation by western blotting. Depletion of either G3BP1 or G3BP2, or both, led to a marked reduction in phospho-ERK levels, indicating that both proteins are required for vIRF1 to activate the MAPK pathway (Fig. 6a and S5a).

Having established that G3BP proteins are functionally necessary, we next asked whether their direct interaction with vIRF1 is also required for signaling. To address this, we took advantage of (-)-epigallocatechin gallate (EGCG), a polyphenol previously shown to interact with and inhibit the G3BPs^47^. We first assessed whether EGCG treatment impairs the vIRF1–G3BP interaction. Indeed, co-immunoprecipitation revealed that EGCG treatment effectively disrupted vIRF1 binding to G3BP1 (Fig. 6b and S5b–d). Importantly, the interaction between vIRF1 and another RNA-dependent interactor, RPL4, was preserved, demonstrating that EGCG selectively interferes with G3BP engagement rather than globally disrupting vIRF1-associated complexes.

To determine whether disrupting the vIRF1–G3BP interaction impairs Ras/MAPK signaling, we first assessed Ras activation using the Raf-RBD pull-down assay. EGCG treatment reduced levels of GTP-bound Ras in BJAB cells expressing WT vIRF1, indicating that Ras activation is compromised when the vIRF1–G3BP interaction is disrupted (Fig. 6c). Consistent with this, phospho-western blotting revealed a parallel reduction in ERK phosphorylation upon EGCG treatment (Fig. 6d). Together, these results demonstrate that intact vIRF1–G3BP interactions are required for activation of the Ras/MAPK cascade, linking RNA-scaffolded complex formation directly to vIRF1-driven MAPK signaling.

We next sought to further test whether vIRF1-driven RAS/MEK/ERK activation depends on an RNA-mediated association with G3BP1. We reasoned that if RNA bridges vIRF1 to its effector G3BP1, then forcing wild-type vIRF1 onto an irrelevant transcript should divert it from its endogenous RNA targets and attenuate downstream signaling, without altering the protein itself. To impose this redirection, we took advantage of the MS2 coat protein (MCP) which binds with high affinity to MS2 RNA hairpins, allowing an MCP-fused protein to be recruited onto any transcript bearing those hairpins^48^. BJAB cells were co-transfected with HA–MCP-tagged wild-type vIRF1 (MCP-vIRF1) and either an MS2 hairpin–tagged *Renilla* luciferase mRNA (MS2-Rluc), which recruits MCP-vIRF1 onto a defined decoy transcript, or an untagged firefly luciferase mRNA (Fluc) that cannot engage MCP and leaves vIRF1 unconstrained. Tethering vIRF1 to the MS2-Rluc decoy reduced both ERK phosphorylation (Fig. S5e) and the vIRF1–G3BP1 interaction (Fig. S5f–i) relative to the Fluc control. These reductions were partial, as anticipated for a competition in which a single ectopic transcript must draw vIRF1 away from the abundant and diverse cellular RNA pool it can otherwise access. These results suggest that vIRF1 activates the Ras/MEK/ERK pathway through an RNA-dependent interaction with G3BP1, and that disrupting this association is sufficient to weaken both the vIRF1-G3BP1 interaction and downstream signaling.

To test whether G3BP proteins contribute to KSHV lytic reactivation, we examined the effects of G3BP1 and G3BP2 depletion on viral gene expression during reactivation in TREx-BCBL1-RNA cells. We silenced G3BP1 and G3BP2 in latently infected TREx-BCBL1-RTA cells and induced reactivation. As expected, depletion of G3BP1 and G3BP2 reduced ERK phosphorylation (Fig. 6e). Consistent with this, GTP-bound Ras was reduced when G3BP was depleted (Fig. 6f). Importantly, RT-qPCR analysis revealed that G3BP knockdown led to a significant reduction in the expression of representative KSHV lytic genes, including ORF57, ORF59 and PAN, reinforcing their importance in facilitating efficient viral gene expression (Fig. 6g–i). Together, these findings establish that G3BP proteins are not only required for Ras/MAPK activation by vIRF1, but are also essential host factors that promote lytic reactivation of KSHV (Fig. 6j).

## Discussion

Kaposi’s sarcoma-associated herpesvirus (KSHV) is a leading cause of AIDS-associated cancers, yet the molecular strategies it employs to reprogram host cells during latency, reactivation, and oncogenesis remain incompletely defined. In particular, how KSHV leverages RNA–protein interactions to manipulate gene expression and signaling has been largely unexplored. To address this, we employed an unbiased, systems-level proteomic approach to map the RNA-binding proteome in KSHV-infected PEL cells. Using XRNAX-based RNA interactome capture during both latent and lytic phases, we comprehensively identified host RBPs and uncovered 14 viral RNA-binding proteins, including 7 not previously known to bind RNA. These findings reveal RNA binding as a pervasive strategy used by KSHV to control the host environment and provide a resource for uncovering new mechanisms of viral replication, immune evasion, and oncogenesis.

In addition to confirming known KSHV-encoded RBPs, our dataset validated previous in vitro observations for four additional viral proteins and uncovered seven viral RBPs not previously recognized to bind RNA. Notably, they all have well-characterized roles unrelated to RNA biology, highlighting the untapped potential to define how RNA binding contributes to their function. For example, K5, a viral E3 ubiquitin ligase that mediates immune evasion by downregulating MHC class I and other components of the immunological synapse^49–51^, emerged as one of the most robust RBPs based on ^32^P-CLIP signal. While the immunological function of K5 has been extensively investigated, its RNA-binding activity introduces an unexpected and intriguing regulatory layer. Interestingly, a growing body of work has identified cellular E3 ligases as an emerging class of RBPs, with roles in gene regulation, RNA stability, and immune modulation^52–55^. Whether K5’s ability to bind RNA influences its substrate specificity, subcellular localization, or interaction with host machinery remains an open question. These findings underscore the broader utility of our RBPome in revealing RNA-dependent functions of multifunctional viral proteins and set the stage for future studies aimed at uncovering new mechanisms of immune evasion and viral pathogenesis.

This dataset not only expands the catalog of KSHV-encoded RBPs but also uncovers new biology critical to the viral lifecycle, exemplified by the discovery of vIRF1 as an RBP that activates Ras/MAPK signaling. MAPK signaling is a central driver of KSHV lytic reactivation, and its activation is essential for the transition from latency to productive replication^38^. While viral proteins such as vGPCR have been shown to stimulate MAPK during latency^56^, our findings reveal a distinct, RNA-dependent mechanism wherein vIRF1 engages the host scaffolding protein G3BP to activate this pathway. G3BP is best known for its role in stress granule formation, but stress granules are notably absent during KSHV infection, likely due to ORF57-mediated sequestration of immunostimulatory dsRNA, leaving G3BP available to participate in alternative functions^57^. G3BP was initially identified as a modulator of Ras activation, and more recently shown to act as a GTPase-activating protein(GAP)-binding protein within the TSC-mTORC1 signaling axis^44,58^. Our data suggest that vIRF1 co-opts G3BP as an RNA-dependent adapter to stimulate MAPK signaling. Whether this interaction is mediated by specific RNAs is not known and future experiments will be directed at determining this.

Although vIRF1 encodes a putative DNA-binding domain, its ability to bind DNA remains unresolved. Unlike cellular IRFs, vIRF1 lacks three of the five conserved tryptophan residues required for sequence-specific DNA binding, and accordingly, it fails to recognize canonical IRF response elements^26,27^. While some studies have proposed that vIRF1 may bind DNA through an alternative or inducible conformation, our functional data argue against a critical role for this activity in KSHV gene regulation^59,60^. Specifically, constitutively active MEK rescues the transcriptional defect of the vIRF1 AAA mutant, indicating that vIRF1-mediated activation of MAPK signaling, rather than direct DNA binding, is key for efficient viral gene expression. This conclusion is further supported by our finding that disrupting the vIRF1-G3BP interaction with EGCG attenuates Ras/MAPK activation, implicating RNA-dependent scaffolding through G3BP, rather than DNA binding, as the primary mechanism by which vIRF1 promotes KSHV reactivation. However, it is now appreciated that transcription factors and chromatin associated proteins can bind RNA, and that this binding can alter their function^2^. Determining if vIRF1 is engaged on chromatin and, if so, the contribution of RNA to this function warrants additional investigation.

These findings uncover an unexpected role for viral protein-RNA binding in modulating a classical signal transduction pathway. Moreover, the discovery that the RNA-binding activity of vIRF1 is critical for MAPK activation in B cells, but not epithelial cells, provides a compelling example of how viruses fine-tune their strategies to the cellular context of infection. Taken together, our study provides a systems-level atlas of KSHV–host RNA–protein interactions and defines a mechanism by which a herpesviral RBP engages RNA to activate cell-intrinsic signaling pathways. The discovery of novel KSHV-encoded RBPs lays the foundation for future studies into their individual contributions to viral replication, immune evasion, and tumorigenesis. More broadly, our work illustrates how RNA functions not only as an informational molecule but also as a structural scaffold that enables viral manipulation of host cell biology.

## Methods

### Cell culture

iSLK-RTA^61^ (a kind gift from Dr. Britt Glaunsinger, University of California, Berkeley), iSLK-BAC16-WT/REV/AAA and HEK293T (ATCC) were grown in Dulbecco’s modified Eagle medium (DMEM; Invitrogen) supplemented with 10% fetal bovine serum (FBS; Invitrogen). TREx-BCBL1-RTA^62^ (a kind gift from Dr. Britt Glaunsinger, University of California, Berkeley), TREx-BCBL1-RTA-GFP/vIRF1, BJAB (a kind gift from Dr. Britt Glaunsinger, University of California, Berkeley), BJAB-BAC16-WT/REV/AAA, BJAB-iRTA-BAC16-REV/AAA and BJAB-ivIRF1-WT/AAA, EBV-negative Akata^37^ (a kind gift from Dr. Benjamin Gewurz, Brigham and Women’s Hospital) cells were maintained in RPMI 1640 medium (Invitrogen) supplemented with 10% FBS (Invitrogen) and 2 mM l-glutamine (Invitrogen). iSLK-BAC16-WT/REV/AAA, BJAB-BAC16-WT/REV/AAA and BJAB-iRTA-BAC16-REV/AAA cells were maintained with 1000 ug/ml Hygromycin B. All cells were maintained with 100 U of penicillin/ml and 100 μg of streptomycin/ml (Invitrogen) at 37 °C under 5% CO_2_. iSLK-BAC16-WT/REV/AAA, TREx-BCBL1-RTA, TREx-BCBL1-RTA-GFP/vIRF1 and BJAB-iRTA-BAC16-REV/AAA cells were lytic reactivated with 1 μg/ml of doxycycline (dox; Fisher Scientific). BJAB-BAC16-WT/REV/AAA and Akata-BAC16-REV/AAA cells were lytic reactivated with 20 ng/ml TPA and 0.1 mM NaB or 1 μg/ml anti-human IgM antibodies (Sigma #10759).

### Protein crosslinked RNA extraction (XRNAX)

Latent or lytic reactivated TREx-BCBL1-RTA cells were washed once with cold PBS and resuspended in cold PBS. UV-crosslinking occurred twice on ice with 300 mJ / cm^2^ at 254 nm wavelength with a CL-1000 UV-crosslinker (UVP). Subsequently, cells were pelleted and stored at −80 °C for up to 2 weeks.

XRNAX experiment was performed in two replicates as described previously^24^. Briefly, 100 million UV crosslinked, or non-crosslinked cells were lysed in 8 mL TRIzol reagent (Thermo) by pipetting up and down to make sure all the cell clumps were disintegrated completely. 1.6 mL chloroform was added to the lysates, mixed by inversion and incubated for 5 min at room temperature. Tubes were centrifuged with 7000 g for 10 min at 4 °C. The aqueous phase was discarded and the interphase was transferred to a 2 mL tube. The interphase was gently washed twice with 1 mL low SDS buffer (Tris-HCl 50 mM, EDTA 1 mM, SDS 0.1%) with retaining the integrity of the interphase flakes. Tubes were spun down with 5000 g for 2 min at room temperature and the supernatant discarded. After the washing, flakes were disintegrated by pipetting into 1 mL of low SDS buffer. Tubes were spun down with 5000 g for 2 min at room temperature and the supernatant was collected as interphase eluate 1. Disintegration of the interphase was repeated with another 1 mL of low SDS buffer, then twice with 1 mL of high SDS buffer (Tris-HCl 50 mM, EDTA 1 mM, SDS 0.5%), yielding approx. 1 mL of interphase eluate 2–4.

The interphase eluates were combined with 60 uL 5 M NaCl, 1 uL Linear Acrylamide (Invitrogen) and 1 mL isopropanol and mixed by inversion. Tubes were spun down for 15 min with 18,000 g at −10 °C. The supernatants were discarded and pellets from all four elutes were combined in 2 mL of 70% ethanol. The combined sample was centrifuged again for 1 min with 18,000 g at room temperature, then the supernatant was discarded and all residual ethanol was removed. 1.8 mL of nuclease-free water was added to the tube and allow the pellet to swell for 1 h on ice with occasional mixing by inversion and eventually dissolved by pipetting.

DNase I treatment was conducted by combining the dissolved interphase with 200 uL 10x NEB DNase I buffer, 2 uL RiboLock RNase inhibitor (Thermo) and 100 uL NEB DNase I and incubated for 60 min at 37 °C with shaking at 700 rpm. Subsequently, the sample was isopropanol precipitated as described above without further addition of Linear acrylamide. Pellets were dissolved in 1 mL nuclease-free water by pipetting.

Two aliquots of 500 mg of XRNAX extract were digested with 10 μg trypsin/LysC in 1 mL buffer containing 50 mM Tris-HCl (pH 8.0), 0.1% SDS and 10 mM DTT for 1 h at 37 °C, 700 rpm shaking. Then CAA was added to a final concentration of 20 mM and digested for another hour. QIAGEN RNeasy Midi Kit was used to purify peptide-crosslinked RNA with modified protocol. 1 mL digest was combined with 3.5 mL buffer RLT in a 15 mL falcon tube, mixed by inversion and heated to 60 °C for 15 min. The sample was cooled to room temperature and combined with 2.5 mL of 100% ethanol. The mixed sample was applied to a RNeasy Midi column by centrifugation with 3000 g for 5 min. The column was washed twice with 2.5 mL buffer RPE and then eluted twice with 250 uL nuclease-free water. All eluates were combined to approx. 900 uL and isopropanol precipitation was done as described above with 1 h incubation at −20 °C and 1 h centrifugation with 18,000 g at −10 °C. The pellet was washed with 70% ethanol and taken up in 60 uL 10 mM Tris-HCl. The sample was heated to 85 °C for 5 min and cooled on ice. Benzonase nuclease (Millipore) was added to the sample and RNA digestion occurred for overnight at 37 °C, 700 rpm shaking.

### Mass Spectrometry

Purifications were digested with trypsin using S-trap (Protifi) according to manufacturer’s recommended protocol. Resulting peptides were analyzed by high resolution data dependent LC-MS/MS on one of two platforms. For one, peptides were autosampled onto a 200 mm by 0.1 mm (Jupiter 3 micron, 300 A), self-packed analytical column coupled directly to a Q-exactive plus mass spectrometer (ThermoFisher) using a nanoelectrospray source and resolved using a 70-minute aqueous to organic gradient. Both the intact masses (MS) and fragmentation patters (MS/MS) of the peptides were collected in a data dependent manner utilizing dynamic exclusion to maximize depth of coverage. Alternatively, data were collected with a DDA-PASEF strategy using a Bruker tims-TOF HT equipped with a NanoElute2 HPLC system. Peptides were separated over a 30-minute aqueous to organic gradient using a PepSep 150 um by 25 cm column. Raw data files generated from the LC-MS/MS instrument were processed using MaxQuant^63^ (1.6.7.0 and 2.6.7.0). The analysis was performed with a peptide-spectrum match (PSM) false discovery rate (FDR) set to 0.01 and a minimum peptide count of 1 to ensure high-confidence peak list generation and accurate identification of peptide-spectrum matches. Searches were performed using a Uniprot/Swissprot database for *Homo sapiens* and HHV-8, with added sequences for Benzonase nuclease (Uniprot #P13717).

### Validation of RNA binding by CLIP

Five million TREx-BCBL1-RTA cells were neon transfected with strep-tagged viral RBP candidates and lytic reactivated for two days. After UV crosslinking as described above, cell pellets were lysed in 1 mL CLIP lysis buffer (Tris-HCl 50 mM pH 7.4, NaCl 100 mM, MgCl_2_ 1 mM, NP40 1%, SDS 0.1%, sodium deoxycholate 0.5%, 1 x EDTA-free protease inhibitor). The samples were spun down for 15 min with 18,000 g at 4 °C and supernatant was transferred to a new tube. 20 units RNase I was added to the sample and incubated at 37 °C for 3 min. 30 uL of MagStrep Strep-Tactin beads (IBA Lifesciences) was used for 2 h of IP at 4 °C on a rotating wheel, followed by two washes with high salt buffer (Tris-HCl 50 mM pH 7.4, NaCl 1 M, EDTA 1 mM, NP-40 1%, SDS 0.1%, sodium deoxycholate 0.5%) and two washes with wash buffer (Tris-HCl 20 mM pH 7.4, MgCl_2_ 10 mM, Tween-20 0.2%). PNK buffer (Tris-HCl 50 mM pH 7.4, NaCl 50 mM, MgCl_2_10 mM). The PNK labeling reaction was then performed on-bead for 15 min at 37 °C by addition of hot PNK mix (PNK buffer supplemented with DTT 5 mM, T4 PNK 1 U/uL, ^32^P-ATP 0.5 uCi/uL), followed by four washes with PNK buffer. Half of the beads were subjected to protease K digestion and TRIzol extraction to purify the bound nucleic acids for Urea-PAGE analysis and the other half of beads were boiled in 1 x SDS-PAGE loading dye for 5 min to extract the RNP and run on SDS-PAGE.

### Plasmids and cloning

pcDNA4/TO-ORF52, K9, ORF11, K5, and ORF59-2XCSTREP^64^ were gifts from Dr. Britt Glaunsinger, University of California, Berkeley. pcDNA4/TO-K9-AAA_161-163_-2XCSTREP was generated by site directed mutagenesis. pSBtet-RP was a gift from Eric Kowarz^65^ (Addgene plasmid # 60497; http://n2t.net/addgene:60497; RRID:Addgene_60497). The C-terminal 2X strep tagged ORF50, WT and mutant vIRF1 (AAA_161-163_) were cloned in pSBtet-RP vector with SfiI restriction site and generated pSBtet-RP-ORF50, pSBtet-RP-vIRF1 WT and AAA plasmids. pLX311 Luciferase was a gift from William Hahn^66^ (Addgene plasmid # 117735; http://n2t.net/addgene:117735; RRID:Addgene_117735). pMS2X24-Rluc and pHA-MCP were a gift from Dr. Britt Glaunsinger, University of California, Berkeley. pHA-MCP-vIRF1 was generated by inserting the WT vIRF1 sequence at the BamHI and XbaI site.

### Viral mutagenesis

vIRF1 AAA mutant (AAA_161-163_) and revertant REV (RRR_161-163_) were generated using the scarless Red recombination system in BAC16 GS1783 *Escherichia coli*^67,68^. The BACs were purified using the Nucleobond BAC 100 kit (Clontech). BAC quality was assessed by digestion with HindIII and KpnI (New England Biolabs).

### Cell line establishment

BJAB cells were microporated using 10 uL Neon transfection system (Invitrogen) with 50 ng of BAC DNA at 1350 v, 40 ms pulse width, and 1 pulse. 24 h post microporation, the cells were selected in medium containing 200 μg/mL hygromycin B. The hygromycin B concentration was increased up to 1000 μg/mL until all non-microporated cells died.

EBV-negative Akata cells were microporated using 10 uL Neon transfection system (Invitrogen) with 50 ng of BAC DNA at 1350 v, 30 ms pulse width, and 1 pulse. 24 h post microporation, the cells were selected in medium containing 200 μg/mL hygromycin B. The hygromycin B concentration was increased up to 1000 μg/mL until all non-microporated cells died.

iSLK-RTA cells were cocultured with HEK293T cells transfected with WT, AAA or REV BAC16 DNA in the ratio of 1:1 with adding 20 ng/ml TPA and 0.3 mM NaB for 4 days. Then the cells were selected with 300 ug/mL Hygromycin, 1 ug/mL puromycin and 250 ug/mL G418 for about one week. The hygromycin B concentration was increased gradually up to 1000 μg/mL until all HEK293T cell were dead.

BJAB and TREx-BCBL1-RTA cells expressing inducible strep tagged WT and AAA mutant vIRF1 were generated using Sleeping Beauty transposon system. BJAB cells were microporated using 10 uL Neon transfection system (Invitrogen) with 25 ng pSB100X and 475 ng of pSBtet-RP-vIRF1 WT or AAA DNA at 1350 v, 40 ms pulse width, and 1 pulse. TREx-BCBL1-RTA cells were microporated using 10 uL Neon transfection system (Invitrogen) with 25 ng pSB100X and 475 ng of pSBtet-RP-vIRF1 DNA at 1600 v, 10 ms pulse width, and three pulses. 24 h post microporation, the cells were selected in medium containing 2 μg/mL puromycin.

### RNA-seq library preparation and sequencing

RNA was extracted from latent and lytic BJAB-BAC16 WT, vIRF1-AAA and vIRF1-REV cells using TRIzol. After DNase I treatment and phenol:chloroform:isoamyl alcohol [25:24:1 (vol/vol)] extraction, the recovered RNA were submitted to the Vanderbilt Technologies for Advanced Genomics (VANTAGE) for paired-end RNA-sequencing libraries preparation and sequencing on a Nova-Seq with 150 cycles.

### siRNA knockdowns

BJAB-BAC16 and BJAB-ivIRF1 cells were microporated using 10 uL Neon transfection system (Invitrogen) with 20 pmol G3BP1/2 siRNA (Supplementary table 1) or MISSION siRNA Universal Negative Control #1 (Sigma) twice at a 48 h interval at 1350 v, 40 ms pulse width, and 1 pulse. TREx-BCBL1-RTA cells were microporated using 10 uL Neon transfection system (Invitrogen) with 20 pmol G3BP1/2 siRNA or MISSION siRNA Universal Negative Control #1 (Sigma) twice at a 48 h interval at 1600 v, 10 ms pulse width, and three pulses. 24 h post the second microporation, cells were treated with dox or TPA and NaB as described above.

### Active ras pull-down

Ten million BJAB-iVIRF1 or TREx-BCBL1-RTA cells with indicated treatment were subjected to GST-Raf-RBD pull-down using active Ras detection kit (Cell signaling #8821) in accordance with the manufacturer’s instructions.

### vIRF1 IP-MS

TREx-BCBL1-RTA with inducible expressed GFP or vIRF1 were treated with 1 ug/mL dox for 2 days. Cell pellets were resuspended in the whole cell lysis buffer (Hepes-KOH pH7.8 50 mM, NaCl 150 mM, EDTA 5 mM, NP40 1%,1 x EDTA-free protease inhibitor) and kept on ice for 10 min. Cell lysates were centrifuged to generate soluble cell extracts, which were then incubated with MagStrep Strep-Tactin XT beads (IBA Lifesciences) at 4 °C overnight. For the RNase -/+ IP, on-beads RNase treatment was performed in buffer D0.15 (Hepes-KOH pH7.8 20 mM, glycerol 15%, KCl 150 mM, EDTA 0.2 mM, NP40 0.1%) with RNase A and RNase III for 2 h at 4 °C. Beads were then washed three times for 5 min at 4 °C in buffer D0.15 and eluted with buffer BXT (IBA Lifesciences). The elutes were subjected to MS analysis as described above.

### Luciferase assays

HEK293T cells were transfected with constructs expressing GFP, wild-type (WT) vIRF1, or vIRF1-AAA mutant, along with an ISRE-luciferase reporter and key activators of the nucleic acid sensing pathway (MAVS, TBK1, IKKε, and IRF3) using PolyJet in vitro DNA transfection reagent (Signagene laboratories). 48 h post transfection cells were collected and used to measure firefly luciferase activity using ONE-Glo EX luciferase assay system (Promega) on a GloMax Discover Microplate Reader (Promega). The data was collected with GloMax Discover software.

### RT-qPCR

Total RNA was isolated with TRIzol (Invitrogen) in accordance with the manufacturer’s instructions. RNA was DNase I (NEB) treated at 37 °C for 20 min and inactivated with EDTA at 70 °C for 10 min. cDNA was synthesized from Dnase-treated RNA with random 6-mer (Integrated DNA Technologies) and M-MLV Reverse Transcriptase (Promega). qPCR was performed using the PowerUp SYBR Green qPCR kit (Thermo Scientific) with appropriate primers (Supplementary Table 1). The qPCR was run on QuantStudio 3 (Thermo Scientific) and data was collected with Quant Studio Design & Analysis Software.

### Western blotting

Whole cell lysates were prepared with lysis buffer (50 mM Tris [pH 7.6], 150 mM NaCl, 0.5% NP-40) and quantified by Bradford assay (BioRad). Equivalent amounts of each sample were resolved by SDS-PAGE, electrotransferred to PVDF membrane (Millipore), and blotted for the indicated proteins. Antibodies: Strep tag (1:500, IBA #2-1517-001), hnRNP C1/2 (1:1000, Abcam #ab133607), GAPDH (1:25,000, Proteintech #60004-1-AP), vIRF1 (1:1000, kindly provided by Dr. Gary S. Hayward, Johns Hopkins University), phospho-ERK1/2 (1:1000, Cell Signaling #4370), ERK1/2 (1:1,00, Cell Signaling #4695), phospho-p38 (1:1000, Cell Signaling #4511), p38 (1:1000, Cell Signaling #8690), phospho-JNK (1:1000, Cell Signaling #4668), JNK (1:1000, Cell Signaling #9252), N-Ras (1:200, Santa Cruz #sc-31), K-Ras-2B (1:1000, Proteintech #16155-1-AP), NCL (1:5000, Proteintech #10556-1-AP), DHX15 (1:2000, Proteintech #12265-1-AP), RPL4 (1:5,000, Proteintech #11302-1-AP), G3BP1 (1:2500, Proteintech #13057-2-AP), G3BP2 (1:2500, Proteintech #16276-1-AP). Primary antibodies were followed by AlexaFluor 680-conjugated secondary antibodies (Life Technologies, goat anti-rabbit #A27042, goat anti-mouse #A28183, 1:10,000) and visualized by Odyssey CLx imaging system (LI-COR) with Image Studio 6.0 software.

### MS data analysis

For the analysis of RNA-binding proteins from XRNAX extracts, the MaxQuant proteinGroups.txt table was filtered to remove entries in ‘reverse’ ‘potential contaminant’ and ‘only identified by site’. Qualitative analysis^69^ was performed using LFQ MS intensities that were normalized to benzonase (P13717, which is constant in all samples). Candidate RBPs were defined from two steps: (i) Include those proteins with intensity signal identified in both experiments in the UV groups but not in the NoUV groups. (ii) For signals identified in both the UV and NoUV groups, select the proteins with intensity ratio ≥ 3 compared with the NoUV groups. After log transformation, R-package limma^70^ (3.54.2) were used for statistical analysis for the processed protein intensities applying the empirical Bayesian method moderated t test with *p* values adjusted for multiple-testing using Benjamini-Hochberg method. High-confidence RBPs were defined as the candidate RBPs with a unique peptide count ≥ 2 in both experiments of UV groups. GO biological process and molecular function analysis was performed using the R packages clusterProfiler^71^. All human genes were used as background and statistical significance was determined using the Fisher’s exact test and Benjamini-Hochberg method to correct for multiple testing. Analysis of enriched PFAM domains was performed using the DAVID Bioinformatics Resource^72^. The statistical significance was determined using the Fisher’s exact test and Bonferroni correction for multiple testing.

For UV crosslinked lytic versus latent analysis, RBPs changed significantly were defined from two steps: (i) Include those proteins with intensity signal identified in both experiments in the latent but not in the lytic groups or in the lytic but not in the latent groups. (ii) For signals identified in both latent and lytic groups, select the proteins with intensity ratio ≥ 3 compared with the other group. After log transformation, R-package limma^70^ (3.54.2) were used for statistical analysis for the processed protein intensities applying the empirical Bayesian method moderated *t*test with *p*-values adjusted for multiple-testing using Benjamini-Hochberg method. The RBPs with p adjust less than 0.05 were selected.

For the analysis of vIRF1 IP-MS, MaxQuant outputs (proteinGroups.txt) were used for downstream relative quantification. Proteins flagged as potential contaminants by MaxQuant were filtered out together with proteins with missing values across all samples. Then missing value was replaced by half the value of the minimum detected signal. After log transformation, R-package limma^70^ (3.54.2) were used for statistical analysis for the processed protein intensities applying the empirical Bayesian method moderated t test with *p* values adjusted for multiple-testing using Benjamini-Hochberg method.

### RNA-seq data analysis

The fastq files containing raw reads were first trimmed using fastp^73^ (0.23.4). Then the trimmed reads were aligned to the human genome (GRCh38.primary) and KSHV genome (GQ994935.1) using STAR^74^ (2.7.3a) before quantifying gene expression using featureCounts against the respective annotations from gencode (https://www.gencodegenes.org/). The differentially expressed genes were defined as adjusted *p*-value < 0.05 and logFC > 1 with edgeR^75^ (3.42.4).

### Quantification and statistical analysis

Using the GraphPad PRISM 10 software, statistical significance was analyzed with two-tailed Welch’s t test for all data presented except mass-spectrometry and bioinformatics analysis. Graphs were plotted with GraphPad PRISM 10 and Affinity Designer (1.10.5).

### Reporting summary

Further information on research design is available in the Nature Portfolio Reporting Summary linked to this article.

## Supplementary information

Supplementary Information

Supplementary Dataset 1

Supplementary Dataset 2

Supplementary Dataset 3

Supplementary Dataset 4

Supplementary Dataset 5

Description of Additional Supplementary Files

Reporting Summary

Transparent Peer Review file

## Data Availability

Mass spectrometry data has been deposited to the ProteomeXchange Consortium under the PRIDE identifier PXD068587. Sequencing data from this study have been deposited in GEO under accession number GSE308077. A reporting summary for this article is available as a Supplementary file. Source Data are provided with this paper.
