# Supplementary material for: RNA binding proteome profiling reveals RNA-dependent activation of oncogenic host signaling during KSHV infection
Source: Nat Commun. 2026 Sep 16;17:10901. doi: 10.1038/s41467-026-77861-x (PMC13677729; doi:10.1038/s41467-026-77861-x)
Supplement: Supplementary file 1 [file 41467_2026_77861_MOESM1_ESM.pdf]

Supplementary Information

RNA binding proteome profiling reveals RNA dependent activation of oncogenic host signaling during KSHV infection.

Zhao et al.

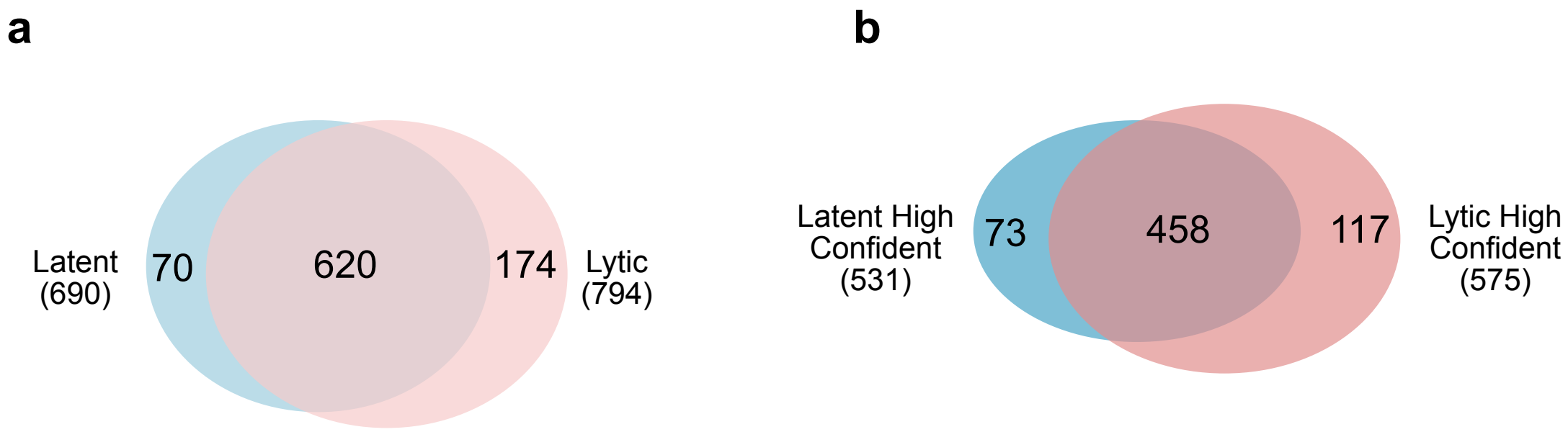

Supplementary Figure 1. Venn diagram showing the overlap of putative RBPs identified from latent and lytic TREx-BCBL1-RTA cells. a, Total number of putative RBPs. **b,** High confidence RBPs that generate >2 unique peptides in both replecates.

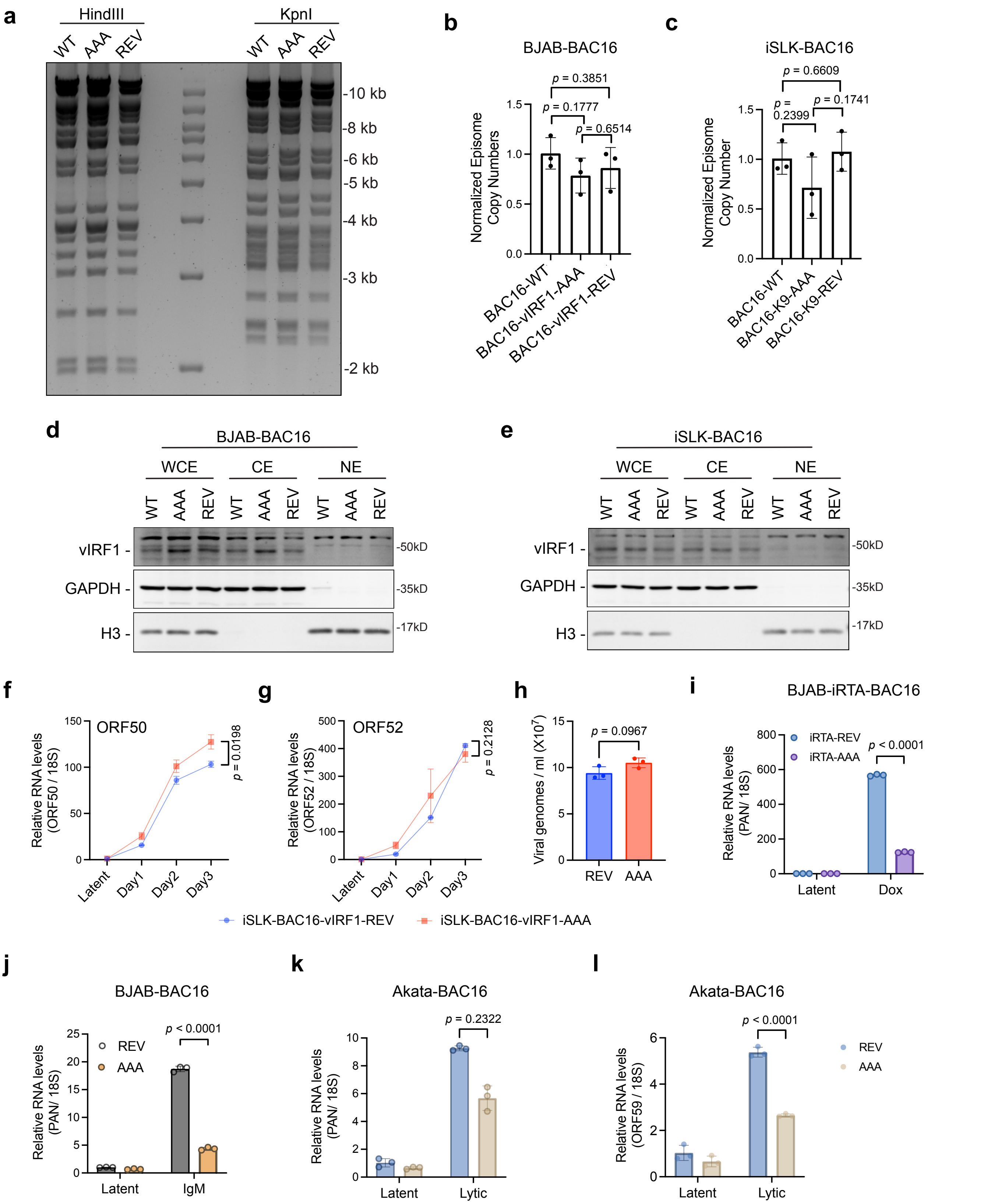

Supplementary Figure 2. vIRF1 RNA binding is dispensable for KSHV lytic reactivation in iSLK cells. a, Representative agarose gel of restriction enzyme digestion of WT vIRF1, mutant AAA and revertant REV BAC16 DNA (n = 3). **b,c,** Quantification of episome number by RT-qPCR in BJAB (**b**) and iSLK (**c**) BAC16 cells. **d,e,** Representative western blot of cellular fraction of BJAB-BAC16 cells reactivated for 1 day with 20 ng/ml TPA and 1mM NaB (**d**) and iSLK-BAC16 cells reactivated with 1 μg/ml dox for 1 day (**e**) (n = 3). **f,g,** RT-qPCR analysis of indicated lytic genes expression in iSLK-BAC16 cells AAA and REV vIRF1. Lytic reactivation was induced by 1 μg/ml dox for up to 3 days. **h,** RT-qPCR quantification of virion production in day 3 samples as in (**f**). **i,** RT-qPCR analysis of PAN expression in BJAB-iRTA-BAC16 cells with AAA and REV vIRF1. **j,** RT-qPCR analysis of PAN expression in BJAB-BAC16 cells treated with anti-human IgM. **k,l,** RT-qPCR analysis of PAN (**k**) and ORF59 (**l**) expression in Akata-BAC16 cells treated with 20 ng/ml TPA and 1mM NaB for 3 days. RT-qPCR data are presented as mean values ± SD (n = 3 biologically independent samples). p-values were determined by the two-tailed Student's t-test. Source data are provided in Source Data file.

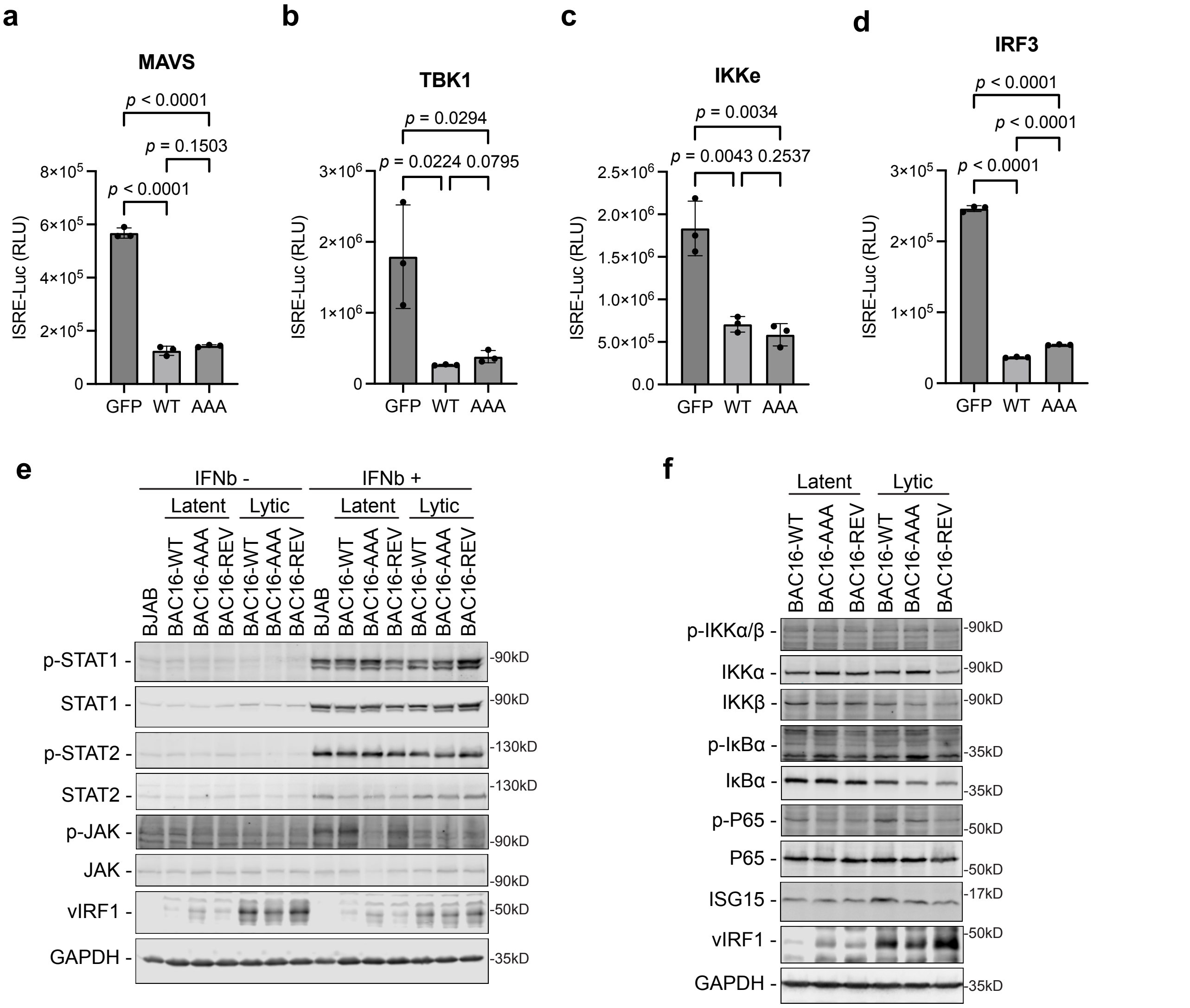

Supplementary Figure 3. Impairment in RNA binding does not alter vIRF1's ability to suppress the interferon (IFN) response. a-d, Luciferase assay of ISRE reporters in HEK293T cells cotransfected of constructs expressing key activators of the nucleic acid sensing pathway and WT or AAA mutant vIRF1. **e,f,** Representative western blot of key factors in IFN (**e**) and NF-κB (**f**) pathways using the protein extracts from BJAB-BAC16 cells with WT, AAA and REV vIRF1 (n = 3). Luciferase data are presented as mean values ± SD (n = 3 biologically independent samples). p-values were determined by the two-tailed Student's t-test. Source data are provided in Source Data file.

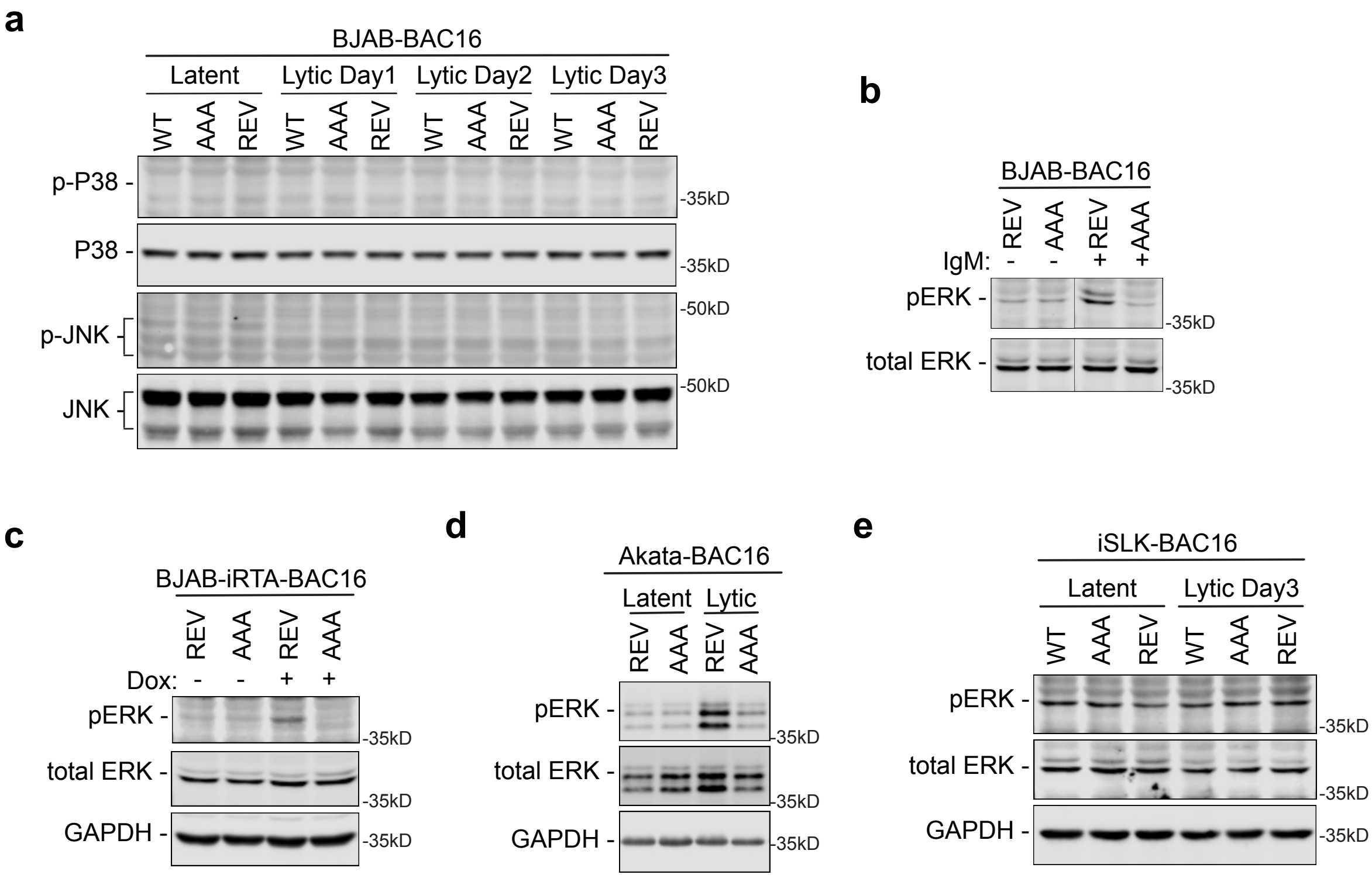

Supplementary Figure 4. vIRF1 RNA binding facilitates ERK activation in B cells but not iSLK cells. a, Representative western blot of protein extracts from BJAB-BAC16 cells reactivated for up to 3 days with 20 ng/ml TPA and 1mM NaB (n = 3). **b,** Representative western blot of protein extracts from BJAB-BAC16 cells reactivated for 3 days with anti-human IgM (n = 3). **c,** Representative western blot of protein extracts from BJAB-iRTA-BAC16 cells reactivated with 1 µg/ml dox for 3 days (n = 3). **d,** Representative western blot of protein extracts from Akata-BAC16 cells reactivated with 20 ng/ml TPA and 1mM NaB for 3 days (n = 3). **e,** Representative western blot of protein extracts from iSLK-RTA-BAC16 cells reactivated with 1 µg/ml dox for 3 days (n = 3). Source data are provided in Source Data file.

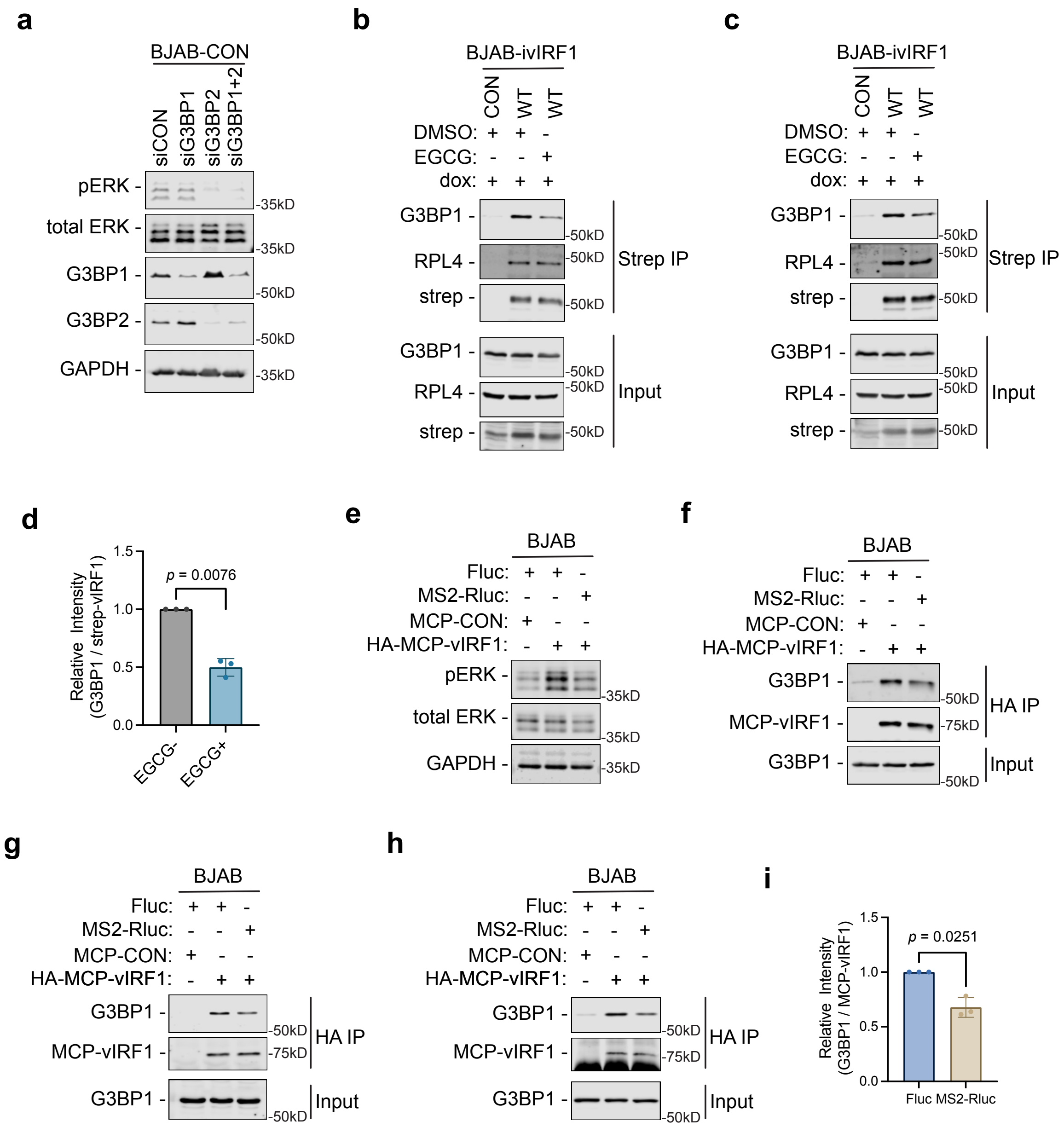

Supplementary Figure 5. An RNA-dependent vIRF1-G3BP interaction facilitates MAPK activation **a,** Representative western blot of BJAB control cells knocking down G3BP proteins with siRNA (n = 3). **b,c,** Biological replicates of figure 6b. **d,** Quantification of vIRF1-bound G3BP1 from b, c and figure 6b. **e,** Representative western blot of protein extracts from BJAB cells neon transfected with constructs as indicated (n = 3). **f,g,h,** Western blot of HA IP of BJAB cells neon transfected with constructs as indicated. **i,** Quantification of vIRF1-bound G3BP1 from f, g and h.Source data are provided in Source Data file.

Supplementary Table 1. List of primers and siRNAs

Oligo and siRNA Name	Sequence 5'-> 3'
ORF57 F	TGGACATTATGAAGGGCATCCTA
ORF57 R	CGGGTTCGGACAATTGCT
PAN F	ATAGGCGACAAAGTGAGGTGGCAT
PAN R	TAACATTGAAAGAGCGCTCCCAGC
ORF52 F	AAATCGAAGCCAGGGTCAGG
ORF52 R	CTCCTCTTCGTCGCCTGTTATTG
18S rRNA F	GTAACCCGTTGAACCCCATT
18SrRNA R	CCATCCAATCGGTAGTAGCG
ORF50 F (Both DOX-inducible and KSHV encoded)	CGCAATGCGTTACGTTGTTG
ORF50 R (Both DOX-inducible and KSHV encoded)	GCCCGGACTGTTGAATCG
ORF50 F (KSHV only)	GAGTCCGGCACACTGTACC
ORF50 R (KSHV only)	AAACTGCCTGGGAAGTTAACG
ORF59 F	ACAGTCACCGTTTGGTCCTC
ORF59 R	TGTACTCGACGCTGGCATAG
siG3BP1	ACAUUUAGAGGAGCCUGUUGCUGAA[dT][dT]
siG3BP2	GAGCUAAAGGAAUUCUUCA[dT][dT]
