# Supplementary material for: RNA binding proteome profiling reveals RNA-dependent activation of oncogenic host signaling during KSHV infection
Source: Nat Commun. 2026 Sep 16;17:10901. doi: 10.1038/s41467-026-77861-x (PMC13677729; doi:10.1038/s41467-026-77861-x)
Supplement: Supplementary file 2 [file 41467_2026_77861_MOESM7_ESM.pdf]

Description of Additional Supplemantary Files

FileName: Supplementary Data 1

Description: XRNAX identified RBPs in latent TREx-BCBL1-RTA cells

FileName: Supplementary Data 2

Description: XRNAX identified RBPs in lytic TREx-BCBL1-RTA cells

FileName: Supplementary Data 3

Description: XRNAX identified RBPs that significantly changed between latent and lytic TREx-BCBL1-RTA cells

FileName: Supplementary Data 4

Description: Strep-vIRF1 IP-MS in lytic TREx-BCBL1-RTA cells: RNase + / -

FileName: Supplementary Data 5

Description: Strep-vIRF1 IP-MS in lytic TREx-BCBL1-RTA cells: AAA / WT
