# Supplementary material for: RNA binding proteome profiling reveals RNA-dependent activation of oncogenic host signaling during KSHV infection
Source: Nat Commun. 2026 Sep 16;17:10901. doi: 10.1038/s41467-026-77861-x (PMC13677729; doi:10.1038/s41467-026-77861-x)
Supplement: Supplementary file 3 [file 41467_2026_77861_MOESM8_ESM.pdf]

nature portfolio

Corresponding author(s): John Karijolich

Last updated by author(s): Aug. 7, 2026

Reporting Summary

Nature Portfolio wishes to improve the reproducibility of the work that we publish. This form provides structure for consistency and transparency in reporting. For further information on Nature Portfolio policies, see our Editorial Policies and the Editorial Policy Checklist.

Statistics

For all statistical analyses, confirm that the following items are present in the figure legend, table legend, main text, or Methods section.

n/a	Confirmed	
☐	☒	The exact sample size (*n*) for each experimental group/condition, given as a discrete number and unit of measurement
☐	☒	A statement on whether measurements were taken from distinct samples or whether the same sample was measured repeatedly
☐	☒	The statistical test(s) used AND whether they are one- or two-sided<br>*Only common tests should be described solely by name; describe more complex techniques in the Methods section.*
☒	☐	A description of all covariates tested
☐	☒	A description of any assumptions or corrections, such as tests of normality and adjustment for multiple comparisons
☐	☒	A full description of the statistical parameters including central tendency (e.g. means) or other basic estimates (e.g. regression coefficient) AND variation (e.g. standard deviation) or associated estimates of uncertainty (e.g. confidence intervals)
☐	☒	For null hypothesis testing, the test statistic (e.g. *F*, *t*, *r*) with confidence intervals, effect sizes, degrees of freedom and *P* value noted<br>*Give P values as exact values whenever suitable.*
☒	☐	For Bayesian analysis, information on the choice of priors and Markov chain Monte Carlo settings
☒	☐	For hierarchical and complex designs, identification of the appropriate level for tests and full reporting of outcomes
☐	☒	Estimates of effect sizes (e.g. Cohen's *d*, Pearson's *r*), indicating how they were calculated

Our web collection on statistics for biologists contains articles on many of the points above.

Software and code

Policy information about availability of computer code

Data collection	The luciferase data was collected with GloMax Discover software. The qPCR data was collected with Quant Studio Design & Analysis Software. The western blot was imaged with Image Studio 6.0 software.
Data analysis	For Mass spectrometry, Raw data files generated from the LC-MS/MS instrument were processed using MaxQuant (1.6.7.0 and 2.6.7.0). The analysis was performed with a peptide-spectrum match (PSM) false discovery rate (FDR) set to 0.01 and a minimum peptide count of 1 to ensure high-confidence peak list generation and accurate identification of peptide-spectrum matches. Searches were performed using a Uniprot/Swissprot database for Homo sapiens and HHV-8, with added sequences for Benzonase nuclease (Uniprot #P13717). For the analysis of RNA-binding proteins from XRNAX extracts, the MaxQuant proteinGroups.txt table was filtered to remove entries in 'reverse' 'potential contaminant' and 'only identified by site'. Qualitative analysis was performed using LFQ MS intensities that were normalized to benzonase (P13717, which is constant in all samples). Candidate RBPs were defined from two steps: (i) Include those proteins with intensity signal identified in both experiments in the UV groups but not in the NoUV groups. (ii) For signals identified in both the UV and NoUV groups, select the proteins with intensity ratio ≥ 3 compared with the NoUV groups. High-confidence RBPs were defined as the candidate RBPs with a unique peptide count ≥ 2 in both experiments of UV groups. GO biological process and molecular function analysis was performed using the R packages clusterProfiler. All human genes were used as background and statistical significance was determined using the Fisher's exact test and Benjamini-Hochberg method to correct for multiple testing. Analysis of enriched PFAM domains was performed using the DAVID Bioinformatics Resource. The statistical significance was determined using the Fisher's exact test and Bonferroni correction for multiple testing. For UV crosslinked lytic versus latent analysis, RBPs changed significantly were defined from two steps: (i) Include those proteins with intensity signal identified in both experiments in the latent but not in the lytic groups or in the lytic but not in the latent groups. (ii) For signals identified in both latent and lytic groups, select the proteins with intensity ratio ≥ 3 compared with the other group. After log transformation, R-package limma (3.54.2) were used for statistical analysis for the processed protein intensities applying the empirical Bayesian method moderated t test with p values adjusted for multiple-testing using Benjamini-Hochberg method. The RBPs with p adjust less than 0.05 were

selected. For the analysis of vIRF1 IP-MS, MaxQuant outputs (proteinGroups.txt) were used for downstream relative quantification. Proteins flagged as potential contaminants by MaxQuant were filtered out together with proteins with missing values across all samples. Then missing value was replaced by half the value of the minimum detected signal. After log transformation, R-package limma (3.54.2) were used for statistical analysis for the processed protein intensities applying the empirical Bayesian method moderated t test with p values adjusted for multiple-testing using Benjamini-Hochberg method.
For RNA-seq data analysis, The fastq files containing raw reads were first trimmed using fastp (0.23.4). Then the trimmed reads were aligned to the human genome (GRCh38.primary) and KSHV genome (GQ994935.1) using STAR (2.7.3a) before quantifying gene expression using featureCounts against the respective annotations from gencode (https://www.gencodegenes.org/). The differentially expressed genes were defined as adjusted p-value < 0.05 and logFC > 1 with edgeR (3.42.4).

For manuscripts utilizing custom algorithms or software that are central to the research but not yet described in published literature, software must be made available to editors and reviewers. We strongly encourage code deposition in a community repository (e.g. GitHub). See the Nature Portfolio guidelines for submitting code & software for further information.

Data

Policy information about availability of data

All manuscripts must include a data availability statement. This statement should provide the following information, where applicable:

- Accession codes, unique identifiers, or web links for publicly available datasets
- A description of any restrictions on data availability
- For clinical datasets or third party data, please ensure that the statement adheres to our policy

Mass spectrometry data has been deposited to the ProteomeXchange Consortium under the PRIDE identifier PXD068587. Sequencing data from this study have been deposited in GEO under the identifier GSE308077 and will be available upon publication.

Research involving human participants, their data, or biological material

Policy information about studies with human participants or human data. See also policy information about sex, gender (identity/presentation), and sexual orientation and race, ethnicity and racism.

Reporting on sex and gender	n/a
Reporting on race, ethnicity, or other socially relevant groupings	n/a
Population characteristics	n/a
Recruitment	n/a
Ethics oversight	n/a

Note that full information on the approval of the study protocol must also be provided in the manuscript.

Field-specific reporting

Please select the one below that is the best fit for your research. If you are not sure, read the appropriate sections before making your selection.

☒ Life sciences ☐ Behavioural & social sciences ☐ Ecological, evolutionary & environmental sciences

For a reference copy of the document with all sections, see nature.com/documents/nr-reporting-summary-flat.pdf

Life sciences study design

All studies must disclose on these points even when the disclosure is negative.

Sample size	No statistical methods were used to determine sample size. We selected sufficient samples sizes to ensure reproducibility of our findings as well as large enough to perform statistical analysis (at least n = 3 unless stated). Our rational for selecting this sample size was based on previous experience with these assays.
Data exclusions	No data were excluded from the analysis
Replication	All experiments were conducted at least 3 times or number indicated in the figure legends.
Randomization	No randomization done for these experiments. Data variability was controlled through inclusion of multiple biological replicates and multiple technical replicates within repeats when necessary.
Blinding	Personnel handling the sequencing of RNA-seq data and the mass sepetrometry were blinded.

Reporting for specific materials, systems and methods

We require information from authors about some types of materials, experimental systems and methods used in many studies. Here, indicate whether each material, system or method listed is relevant to your study. If you are not sure if a list item applies to your research, read the appropriate section before selecting a response.

Materials & experimental systems

n/a	Involved in the study
☐	☒ Antibodies
☐	☒ Eukaryotic cell lines
☒	☐ Palaeontology and archaeology
☒	☐ Animals and other organisms
☒	☐ Clinical data
☒	☐ Dual use research of concern
☒	☐ Plants

Methods

n/a	Involved in the study
☒	☐ ChIP-seq
☒	☐ Flow cytometry
☒	☐ MRI-based neuroimaging

Antibodies

Antibodies used	Antibodies: Strep tag (1:500, IBA #2-1517-001), hnRNP C1/2 (1:1,000, Abcam #ab133607), GAPDH (1:25,000, Proteintech #60004-1-AP), vIRF1 (1:1,000, kindly provided by Dr. Gary S. Hayward, Johns Hopkins University), phospho-ERK1/2 (1:1,000, Cell Signaling #4370), ERK1/2 (1:1,000, Cell Signaling #4695), phospho-p38 (1:1,000, Cell Signaling #4511), p38 (1:1,000, Cell Signaling #8690), phosphor-JNK (1:1,000, Cell Signaling #4668), JNK (1:1,000, Cell Signaling #9252), N-Ras (1:200, Santa Cruz #sc-31), K-Ras-2B (1:1,000, Proteintech #16155-1-AP), NCL (1:5,000, Proteintech #10556-1-AP), DHX15 (1:2,000, Proteintech #12265-1-AP), RPL4 (1:5,000, Proteintech #11302-1-AP), G3BP1 (1:2,500, Proteintech #13057-2-AP), G3BP2 (1:2,500, Proteintech #16276-1-AP).
Validation	Antibodies hnRNP C1/2 (Abcam #ab133607), GAPDH (Proteintech #60004-1-AP), phospho-ERK1/2 (Cell Signaling #4370), ERK1/2 (Cell Signaling #4695), phospho-p38 (Cell Signaling #4511), p38 (Cell Signaling #8690), phosphor-JNK (Cell Signaling #4668), JNK (Cell Signaling #9252), N-Ras (Santa Cruz #sc-31), K-Ras-2B (Proteintech #16155-1-AP), NCL (Proteintech #10556-1-AP), DHX15 (Proteintech #12265-1-AP), RPL4 (Proteintech #11302-1-AP), G3BP1 (Proteintech #13057-2-AP), G3BP2 (Proteintech #16276-1-AP) were validated on the manufacturer website for western blot and IP as indicated.

Eukaryotic cell lines

Policy information about cell lines and Sex and Gender in Research

Cell line source(s)	iSLK-RTA, TREx-BCBL1-RTA and BJAB cells were provided by Dr. Britt Glaunsinger, University of California, Berkeley. HEK293T cells were from ATCC. iSLK-BAC16-WT/REV/AAA, TREx-BCBL1-RTA-GFP/vIRF1, BJAB-BAC16-WT/REV/AAA, BAJB-iRTA-BAC16-REV/AAA, BJAB-ivIRF1-WT/AAA, Akata-BAC16-REV/AAA cells were generated in this study.
Authentication	iSLK-RTA cells were generated in this following paper: Myoung, J. & Ganem, D. Generation of a doxycycline-inducible KSHV producer cell line of endothelial origin: Maintenance of tight latency with efficient reactivation upon induction. J. Virol. Methods 174, 12–21 (2011). TREx-BCBL1-RTA cells were generated in this following paper Nakamura, H. et al. Global Changes in Kaposi's Sarcoma-Associated Virus Gene Expression Patterns following Expression of a Tetracycline-Inducible Rta Transactivator. J. Virol. 77, 4205–4220 (2003). EBV-negative Akata cells were isolated in this following paper Shimizu, N., Tanabe-Tochikura, A., Kuroiwa, Y. & Takada, K. Isolation of Epstein-Barr virus (EBV)-negative cell clones from the EBV-positive Burkitt's lymphoma (BL) line Akata: malignant phenotypes of BL cells are dependent on EBV. J. Virol. 68, 6069–6073 (1994). iSLK-BAC16-WT/REV/AAA, TREx-BCBL1-RTA-GFP/vIRF1, BJAB-BAC16-WT/REV/AAA, BAJB-iRTA-BAC16-REV/AAA, BJAB-ivIRF1-WT/AAA and Akata-BAC16-REV/AAA cells were generated in this study. They are not further autheticated.
Mycoplasma contamination	All cell lines used in this study were mycoplasma free. Cell lines were routinely tested for mycoplasma by PCR.
Commonly misidentified lines (See ICLAC register)	No commonly misidentified cell lines were used in the study.

Plants

Seed stocks	n/a
Novel plant genotypes	n/a
Authentication	n/a
