# Supplementary material for: RNA binding proteome profiling reveals RNA-dependent activation of oncogenic host signaling during KSHV infection
Source: Nat Commun. 2026 Sep 16;17:10901. doi: 10.1038/s41467-026-77861-x (PMC13677729; doi:10.1038/s41467-026-77861-x)
Supplement: Supplementary file 4 [file 41467_2026_77861_MOESM9_ESM.pdf]

nature portfolio

Peer Review File

RNA binding proteome profiling reveals RNA dependent activation of oncogenic host signaling during KSHV infection

Corresponding Author: Dr John Karijolich

This file contains all reviewer reports in order by version, followed by all author rebuttals in order by version.

Version 0:

Reviewer comments:

Reviewer #1

(Remarks to the Author)
In this manuscript, Zhao et al are using proteomics to identify novel protein-RNA interactions in the context of infection by the herpesvirus KSHV. They have identified 7 viral proteins with previously unknown RNA binding ability, and they further explore one: vIRF1. They show that this viral protein can activate the RAS/MAPK pathway via its interaction with G3BP and further show that vIRF1 promotes viral replication via its predicted RNA binding domain. These findings are novel and prompts further investigations into the importance of RNA interactions during viral infection. However, there are inconsistencies in the data, lack of experimental details and some missing controls, and some of the claims do not match the data. Overall, while this study is interesting, perhaps of limited scope, but underscoring the importance of RNA-protein interactions.

Major comments:
- on line 70, the authors provide us with a list of KSHV proteins known to "directly interact with RNA" and further down on line 75-76, a list of viral proteins with known RNA binding capability in vitro. It seems that some of these viral proteins also have in vivo confirmation of their RNA binding potential (LANA: PMID: 24257619; ORF10: PMID: 27832591; ORF59: PMID: 21957289) and ORF26 was also shown to bind PAN RNA (PMID: 21957289)

- line 123: "identified 690 and 794 candidate RBPs in latent and lytic reactivated cells" – in the figure legend, the authors mention that their high confidence list is made of RBP with 2 unique peptides which is by itself a low threshold for "high confidence". It would be better to only mention these "high confidence" numbers and it would also be more relevant in the Venn diagram (fig 1c, 1d) to show the overlap of RBP between latent and lytic samples instead of high/low confidence hits.

- the authors should explain why not all viral controls are recapitulated in their XRNAX approach or at least discuss the limitations of their approach.

- the figure legend or method section does not mention the number of biological replicates for the XRNAX – MS approach (based on the tables, it seems that the number of replicates is only 2 but it is not mentioned in the text).

- if the VIRF1 AAA mutant leads to "profound defect in viral gene expression relative to the Revertant" (line 203) – how did the authors manage to establish this BJAB cell line? It is not mentioned anywhere whether they complemented vIRF1 expression in the co-culture.

- figure 3a and 3b, a loading control is missing to fully interpret the "WB input" blot

- in figure 3c, vIRF1 AAA mutant appear to be less expressed or less stable (fainter band): the authors do not discuss this. Could their defect in lytic replication simple be because less vIRF1 is expressed? Same question for the "striking loss of phospho-ERK1/2" (line 255) with the vIRF1 AAA mutant at day 3 post reactivation (Fig 4a) where vIRF1 AAA is much less expressed at this time point. The authors need to test that increasing the expression of vIRF1 AAA does not rescue ERK phosphorylation.

- in Figure S1b-f, the authors show that viRF1 WT and the AAA mutant infection led to similar level of viral replication, viral

gene expression and viral copy number. Yet, the biggest conclusion of the paper is that this newly identified RNA binding domain is critical for KSHV life cycle. This is inconsistent. If vIRF1's role is so critical, why would it be different in the iSLK background?

- there is a surprising shadow/blurry area in the vIRF1 strep staining in fig 4h (4th lane) – the authors need to provide a better replicate image

- Figure 5: unclear why the authors decided to switch from their BJAB model to use TREX-BCBL1-RTA cells here. These cells are PEL derived and therefore endogenously express vIRF1 from the viral episome. Overexpression of the WT or AAA may interfere with the endogenous expression.

-Figure 6a: there is a missing control: knocking down the G3BPs in the "CON" condition where no vIRF1 is expressed. Otherwise, how do the authors know that KD G3BP does not by itself activate pERK?

-line 347: "EGCG treatment effectively disrupted vIRF1 binding to G3BP1" the effect on the blot is fairly minor – this should be confirmed by a reverse IP.

Minor Comments:
-supplementary table 1 is called out in the text as the qPCR primer table but is missing and instead, the mass spec tables are provided as supplementary tables 1-4 but are not called in the text.

-line 172: references seem off and the font is different

-fig 3a: there is no details on how the vIRF1 mutants were constructed (plasmid backbone, position of the tag etc.).

- the supplementary table showing the data from the XRNAX-MS result is missing a column with the protein name, which would make it a lot easier for the reader.

- in fig 3i: the REV virus seems to also have important deviations from the WT – can the authors comment on that

- Supplementary Figure 2e is missing a loading control and both S2e and S2f should show vIRF1 expression levels

- in the figure legends, some bar graphs are described as mean + SEM and others as mean + SD. Consistency would be better.

- the qPCR data in fig 4 do not mention what was used for normalization

- fig 4e should show a vIRF1 band

-Fig 5e, f: it would be easier for the reader if the proteins were ordered in the same way between these two panels.

Reviewer #2

(Remarks to the Author)
In this paper, the Karijolich group uses a novel discovery-based approach to identify new KSHV RBPs. They report that seven KSHV proteins that were previously not implicated in RNA-binding are bona fide RBPs (ORF8, ORF39, K9, K5, ORF61, K2, ORF11). Next, they explore the potential functions for RNA-binding for K9/vIRF1. They define its RNA binding region and show that mutation of its RNA binding domain abrogates viral reactivation in a cell type-specific manor. Mechanistically, they suggest that its RNA binding activity is essential for RNA-dependent interactions with G3BPs which then modulate ERK phosphorylation and signaling. Overall, the data are clear, rigorous, and well presented. I have only three minor/moderate concerns.

1. vIRF1 has been reported to be primarily nuclear (Seo et al. 2002), with some cytoplasmic signal. Mutation of the RBD in vIRF1 could, in principle, change its localization, particularly since nuclear localization signals tend to be Arg-rich. As such, it becomes important that the localization of their WT and AAA mutant proteins be tested. This is also important for their model which proposes a cytoplasmic mechanism.

2. Throughout the paper, they over-interpret their AAA mutant phenotypes. Each phenotype is interpreted as being due to RNA-binding. While I think their data identifying and characterizing the binding are strong, it doesn't mean that every phenotype that arises from that mutant is due to that activity. Additional characteristics like localization, folding, or protein-protein interactions could all be altered by that mutation. In some cases (e.g., Fig 5a/b) they have orthogonal approaches, to bolster that interpretation. However, in many cases they do not so they cannot exclude these other models. To be clear, I'm not suggesting that they demonstrate that these phenotypes are specifically due to RNA binding. They simply need to interpret that their observations appropriately by saying their data are "consistent with" an RNA binding defect, or "depend on the RNA binding motif". Currently, it seems they interpret every AAA deficiency as "proof" of the importance of RNA binding for that phenotype. Even though I completely agree with the authors that it represents the simplest hypothesis, such

statements over-interpret the data.

3. Similarly, more caution should be taken in interpreting the RNA-dependent interactions in Fig 5. Since vIRF1 binds RNA, it is not surprising that it co-purifies on other abundant RBPs in an RNA-dependent fashion. Whether or not these interactions occur in cells is not demonstrated by these experiments. Again, they do not need to prove this, but they should concede to this limitation.

Reviewer #3

(Remarks to the Author)
This study applies XRNAX to KSHV-infected PEL cells, defining host and viral RNA-binding proteomes under latent and lytic conditions and nominating 14 viral RBPs, 7 of which are newly proposed. The follow-up centers on vIRF1 (K9): the authors generate a vIRF1-AAA mutant that shows reduced RNA binding compared to wild-type. This mutant displays B-cell–specific defects in lytic gene expression and virion production that trace to impaired RAS/MEK/ERK activation and an RNA-sensitive interaction with G3BP1/2. The work is novel, and the dataset will be a useful resource for the community. The G3BP axis is biologically plausible given literature on RNA-driven G3BP conformational switching/condensation and established G3BP signaling roles. Nevertheless, the central mechanistic claim, strict RNA-dependence of vIRF1-driven MAPK activation, relies largely on a triple-alanine substitution that could also perturb protein–protein interfaces. The manuscript would benefit from orthogonal, non-mutational tests and a few areas of statistical and validation rigor. Addressing the points below would considerably strengthen the paper's impact.
Major comments
1- the conclusion that vIRF1 activates RAS/MEK/ERK in an RNA-dependent manner rests predominantly on the RRR→AAA mutant and RNase-sensitive interactions. Since basic-patch mutations can also perturb protein–protein interfaces and local electrostatics, it is important to include non-mutational evidence of RNA-dependence in BJAB: e.g., identify the vIRF1-bound RNA(s) (RIP-qPCR/eCLIP), RNA decoy/mimic competition that reduces vIRF1–G3BP co-IP
2- Using a semi-quantitative, rule-based approach is reasonable only for proteins with complete absence across replicates in one condition (true "on/off" behavior), but for proteins quantified in both conditions, inferential statistics should be applied rather than a hard ratio cutoff. The authors already use limma for the IP-MS datasets (p.29–30), so the same moderated t-test framework (with BH FDR control) should be applied to the XRNAX UV vs No-UV comparisons as well. Also in order to reveal the magnitude of RBPome changes during reactivation direct comparison between lytic(+UV) vs. latent(+UV) samples is important.
3- The B-cell specificity angle is compelling but currently rests only on two lines BJAB vs iSLK that show opposite phenotype. It is important to include more B-cell model (a distinct PEL line or primary B cells if feasible).
Minor points:
1- Statistics reporting inconsistencies & replication clarity: Figure legends state mean ± SD (and elsewhere mean ± SEM) with Student's t-test or one-way ANOVA and the n per experiment aren't uniformly stated.
2- "Two aliquots of 500 mg of XRNAX extract were digested with 10 mg trypsin/LysC" line 493. this is supposed to be ug?
3- The current 32P-CLIP validation set is skewed toward previously known or partially characterized viral RBPs, while only a minority of the newly proposed candidates were tested. Please elaborate the criteria used to choose proteins for 32P-CLIP.
Typos:
"well-stablished PAMP" → well-established (p.3, line 41–42).
"Qualitive analysis" → Qualitative (p.6, line 121–122).
"typically rang from 300 to over 2,000" → range (p.6, line 121–123).
"bond nucleic acids" → bound nucleic acids (Methods, p.24–25).
"laborotaries" → laboratories (Methods, Luciferase assays, p.27, line ~600).
"Quantification and statical analysis" → Quantification and statistical analysis (p.30, line 667).
"seleced" → selected (Methods, cell line selection, p.25–26).
"BAJB" and "BAJB-iRTA" → BJAB (multiple instances on p.20–26).
"phosphor-JNK" → phospho-JNK (Antibody list, p.28, lines 618–621).
"p32-CLiP" → consistently 32P-CLIP (Fig. 3 legend; Fig. 2 panels).
"contitutive active ERK" (Figure text overlays) → constitutive.

Version 1:

Reviewer comments:

Reviewer #1

(Remarks to the Author)

Reviewer #2

(Remarks to the Author)
The authors have appropriately addressed all of my concerns.

Open Access This Peer Review File is licensed under a Creative Commons Attribution 4.0 International License, which permits use, sharing, adaptation, distribution and reproduction in any medium or format, as long as you give appropriate credit to the original author(s) and the source, provide a link to the Creative Commons license, and indicate if changes were made.
In cases where reviewers are anonymous, credit should be given to 'Anonymous Referee' and the source.
The images or other third party material in this Peer Review File are included in the article's Creative Commons license, unless indicated otherwise in a credit line to the material. If material is not included in the article's Creative Commons license and your intended use is not permitted by statutory regulation or exceeds the permitted use, you will need to obtain permission directly from the copyright holder.
To view a copy of this license, visit https://creativecommons.org/licenses/by/4.0/

Response to Reviewers

We thank the reviewers for their thorough and constructive evaluation. Each reviewer comment is reproduced in full, followed by our point-by-point response. Newly added or revised manuscript text is shown in italics within the responses.

Reviewer #1

Comment: In this manuscript, Zhao et al are using proteomics to identify novel protein-RNA interactions in the context of infection by the herpesvirus KSHV. They have identified 7 viral proteins with previously unknown RNA binding ability, and they further explore one: vIRF1. They show that this viral protein can activate the RAS/MAPK pathway via its interaction with G3BP and further show that vIRF1 promotes viral replication via its predicted RNA binding domain. These findings are novel and prompts further investigations into the importance of RNA interactions during viral infection. However, there are inconsistencies in the data, lack of experimental details and some missing controls, and some of the claims do not match the data. Overall, while this study is interesting, perhaps of limited scope, but underscoring the importance of RNA-protein interactions.

- on line 70, the authors provide us with a list of KSHV proteins known to “directly interact with RNA” and further down on line 75-76, a list of viral proteins with known RNA binding capability in vitro. It seems that some of these viral proteins also have in vivo confirmation of their RNA binding potential (LANA: PMID: 24257619; ORF10: PMID: 27832591; ORF59: PMID: 21957289) and ORF26 was also shown to bind PAN RNA (PMID: 21957289)

Response: We thank the reviewer for this helpful observation. We have added ORF26 to line 76 and revised the wording to clarify the nature of these interactions: “*In addition, ORF10, ORF26, ORF52, ORF59, and LANA have been reported to associate with RNA in vitro; however, their direct RNA-binding activity in vivo remains undetermined.*”

We agree that the cited studies show ORF10, ORF26, ORF59, and LANA associate with RNA in cells, and our distinction here is deliberate. The cited work relied on co-immunoprecipitation without cross-linking or with formaldehyde cross-linking, approaches that capture both direct and indirect complexes and therefore cannot resolve whether a given interaction is direct. Demonstrating direct binding *in vivo* requires covalent capture by UV cross-linking (e.g., CLIP-based methods), which has not been reported for these proteins. We have therefore retained the distinction between in-cell RNA association and confirmed direct RNA binding *in vivo*.

Comment: line 123: “identified 690 and 794 candidate RBPs in latent and lytic reactivated cells” – in the figure legend, the authors mention that their high confidence list is made of RBP with 2 unique peptides which is by itself a low threshold for “high confidence”. It

would be better to only mention these “high confidence” numbers and it would also be more relevant in the Venn diagram (fig 1c, 1d) to show the overlap of RBP between latent and lytic samples instead of high/low confidence hits.

Response: We thank the reviewer for this suggestion. Our mass spectrometry analysis follows the established Nature Protocols framework (PMID 29431736), and Figures 1c and 1d have been formatted accordingly. To provide a clearer overview of the dataset, we have added the Venn diagram showing the overlap of RBPs between the latent and lytic samples as Fig. S1. The text at line 125 has been updated to reflect these changes.

Comment: the authors should explain why not all viral controls are recapitulated in their XRNAX approach or at least discuss the limitations of their approach.

Response: We have added a discussion at lines 149 - 156 explaining why XRNAX did not capture ORF26 and ORF37: “*Intriguingly, we did not capture ORF37, which functions as an endonuclease that drives global mRNA degradation. We hypothesize the transient nature of the nuclease–substrate interaction likely makes it difficult to capture by XRNAX. Regarding for ORF26, we speculate that its reported interaction with RNA in the in vitro affinity purification assays might be mediated by other protein within cells, as the XRNAX method is specifically designed to capture only direct RNA-protein interactions via covalent UV cross-linking.*”

Comment: the figure legend or method section does not mention the number of biological replicates for the XRNAX – MS approach (based on the tables, it seems that the number of replicates is only 2 but it is not mentioned in the text).

Response: We thank the reviewer for noting this. We have clarified at line 526 that the XRNAX experiment was performed in two replicates per sample: “*The XRNAX experiment was performed in two replicates as described previously.*”

Comment: if the VIRF1 AAA mutant leads to “profound defect in viral gene expression relative to the Revertant” (line 203) – how did the authors manage to establish this BJAB cell line? It is not mentioned anywhere whether they complemented vIRF1 expression in the co-culture.

Response: The BJAB-BAC16-vIRF1-AAA cell line was generated and maintained in latency using standard electroporation and antibiotic selection, as described in the Methods (“Cell line establishment”); complementation with WT vIRF1 is not required. As shown in Fig. 3e–i, the vIRF1-AAA mutant primarily impairs lytic, not latent, viral gene expression. The latent BJAB-BAC16-vIRF1-AAA cells therefore remain viable and are maintained under normal culture conditions.

Comment: figure 3a and 3b, a loading control is missing to fully interpret the “WB input” blot

Response: GAPDH input blots have been added to Figures 3a and 3c.

Comment: in figure 3c, vIRF1 AAA mutant appear to be less expressed or less stable (fainter band): the authors do not discuss this. Could their defect in lytic replication simple be because less vIRF1 is expressed? Same question for the "striking loss of phospho-ERK1/2" (line 255) with the vIRF1 AAA mutant at day 3 post reactivation (Fig 4a) where vIRF1 AAA is much less expressed at this time point. The authors need to test that increasing the expression of vIRF1 AAA does not rescue ERK phosphorylation.

Response: In Fig. 3c, TREx-BCBL1-RTA cells were electroporated with Strep-tagged WT or mutant vIRF1 and subjected to CLIP two days after reactivation (Methods, line 599); inputs were detected with a Strep antibody. The vIRF1-AAA band is comparable in intensity to WT. Variation largely reflects differences in electroporation efficiency, input band intensity is not a reliable readout of protein expression or stability.

Regarding Fig. 4a, the lower vIRF1 level in AAA-virus cells at day 3 is the expected consequence of the mutant's profound lytic defect (Fig. 3e–g, i). To directly test whether the AAA defect reflects reduced expression, we used BJAB cells expressing dox-inducible Strep-tagged WT or AAA vIRF1 (Fig. 4h). Under dox induction, WT and AAA reached comparable levels, and AAA was in fact slightly higher, yet ERK phosphorylation was strongly induced only by WT. Together, these data establish that the lytic replication and ERK phosphorylation defects of the AAA virus are not caused by reduced vIRF1 expression.

Comment: in Figure S1b-f, the authors show that viRF1 WT and the AAA mutant infection led to similar level of viral replication, viral gene expression and viral copy number. Yet, the biggest conclusion of the paper is that this newly identified RNA binding domain is critical for KSHV life cycle. This is inconsistent. If vIRF1's role is so critical, why would it be different in the iSLK background?

Response: This difference is expected and, we would argue, reinforces rather than undermines our central conclusion. Although widely used in KSHV research for historical reasons, SLK cells are not of KS origin, and are in fact a cross-contaminant derived from a renal carcinoma epithelial line and lack biological relevance to the native KSHV reservoir. Their baseline signaling, receptor expression, and interferon-stimulated gene (ISG) pathways differ markedly from B lymphocytes or primary endothelial cells. Additionally, it is well established KSHV itself behaves differently across cell types. For example, maintaining spontaneous lytic reactivation in B cells while exhibiting artificially tight latency in SLK cells. This points to fundamental differences to viral infection in epithelial versus a B cells. Consistent with this, we and others have shown that transcription start-site usage and ORF utilization for viral genes vary between B cells and epithelial cells (PMID 11119611, 31188901, 38922687).

Accordingly, our conclusion, reflected in the section subtitle “vIRF1 RNA-binding is critical for KSHV lytic reactivation in B cells”, is specific to the B-cell context, and the absence of a lytic defect in iSLK-BAC16 vIRF1-AAA is fully consistent with it.

Comment: there is a surprising shadow/blurry area in the vIRF1 strep staining in fig 4h (4th lane) – the authors need to provide a better replicate image

Response: We have replaced the panel with the original, unadjusted image, which shows no blurring, in the revised Fig. 4h.

Comment: Figure 5: unclear why the authors decided to switch from their BJAB model to use TREX-BCBL1-RTA cells here. These cells are PEL derived and therefore endogenously express vIRF1 from the viral episome. Overexpression of the WT or AAA may interfere with the endogenous expression.

Response: Because the endogenous vIRF1 antibody is not suitable for immunoprecipitation, we expressed strep-tagged WT or AAA vIRF1 in TREx-BCBL1-RTA cells for co-immunoprecipitation and mass spectrometry. This is a common approach in the field. While the mass spectrometry was performed in TREx-BCBL1-RTA cells, we confirmed the key interactions by Western blot in both TREx-BCBL1-RTA and BJAB cells, indicating that the RNA-dependent vIRF1 interactions is not restricted to TREx-BCBL1-RTA cells.

Comment: Figure 6a: there is a missing control: knocking down the G3BPs in the “CON” condition where no vIRF1 is expressed. Otherwise, how do the authors know that KD G3BP does not by itself activate pERK?

Response: We have added the requested experiment. We depleted G3BP1 and G3BP2, individually and together, in BJAB-iCON cells and monitored ERK phosphorylation. As shown in Fig. S5a, G3BP knockdown alone did not induce ERK activation.

Comment: -line 347: “EGCG treatment effectively disrupted vIRF1 binding to G3BP1” the effect on the blot is fairly minor – this should be confirmed by a reverse IP.

Response: We have repeated the EGCG treatment and strep-vIRF1 IP three times (Fig. 6b, S5c, d) and quantified the G3BP1:Strep-vIRF1 ratio with statistical testing. As shown in Fig. S5d, EGCG treatment reduced vIRF1-bound G3BP1 by ~50% on average ($p = 0.0076$).

Minor Comments:

Comment: supplementary table 1 is called out in the text as the qPCR primer table but is missing and instead, the mass spec tables are provided as supplementary tables 1-4 but are not called in the text.

Response: We thank the reviewer for catching this. Supplementary Table 1 (primer list) is now included in the supplementary files, and the updated Supplementary Data 1–5 (mass spectrometry tables) are now cited in the text at lines 124, 127, 350, and 354.

Comment: -line 172: references seem off and the font is different

Response: The reference formatting and font at line 172 have been corrected.

Comment: -fig 3a: there is no details on how the vIRF1 mutants were constructed (plasmid backbone, position of the tag etc.).

Response: We have added a “Plasmids and cloning” section to the Methods at line 618, detailing construction of the vIRF1 mutants.

Comment: - the supplementary table showing the data from the XRNAX-MS result is missing a column with the protein name, which would make it a lot easier for the reader.

Response: A gene-name column has been added to Supplementary Data 1 and 2.

Comment: - in fig 3i: the REV virus seems to also have important deviations from the WT – can the authors comment on that

Response: We have added the following clarification at line 226: “*Total RNA sequencing of latent and lytic reactivated BJAB-BAC16 WT, vIRF1-AAA and vIRF1-REV cells indicated that latent gene expression was comparable between all three conditions, with only minor variations observable. In contrast, lytic reactivated BAC16 vIRF1-AAA had a general and profound defect in viral gene expression when compared to BAC16 WT and vIRF1-REV (Figure 3i).*”

Comment: - Supplementary Figure 2e is missing a loading control and both S2e and S2f should show vIRF1 expression levels

Response: A GAPDH loading control has been added to Fig. S3e, and vIRF1 blots have been added to Fig. S3e and S3f.

Comment: - in the figure legends, some bar graphs are described as mean + SEM and others as mean + SD. Consistency would be better.

Response: All bar graphs are now presented as mean ± SD from three biologically independent samples, with p-values determined by two-tailed Student’s t-test. The figure legends have been updated accordingly.

Comment: - the qPCR data in fig 4 do not mention what was used for normalization.

Response: All RT-qPCR data in Figure 4 are normalized to 18S rRNA. The y-axes in Fig. 4c, d, f, and g have been updated to indicate this.

Comment: fig 4e should show a vIRF1 band.

Response: A vIRF1 blot has been added to Fig. 4e.

Comment: -Fig 5e, f: it would be easier for the reader if the proteins were ordered in the same way between these two panels.

Response: The blots in Fig. 5e and f have been reordered to match.

Reviewer #2

Comment: In this paper, the Karijolich group uses a novel discovery-based approach to identify new KSHV RBPs. They report that seven KSHV proteins that were previously not implicated in RNA-binding are bona fide RBPs (ORF8, ORF39, K9, K5, ORF61, K2, ORF11). Next, they explore the potential functions for RNA-binding for K9/vIRF1. They define its RNA binding region and show that mutation of its RNA binding domain abrogates viral reactivation in a cell type-specific manor. Mechanistically, they suggest that its RNA binding activity is essential for RNA-dependent interactions with G3BPs which then modulate ERK phosphorylation and signaling. Overall, the data are clear, rigorous, and well presented. I have only three minor/moderate concerns.

Response: We sincerely thank the reviewer for the positive assessment and constructive comments, which we address point by point below.

Comment: 1. vIRF1 has been reported to be primarily nuclear (Seo et al. 2002), with some cytoplasmic signal. Mutation of the RBD in vIRF1 could, in principle, change its localization, particularly since nuclear localization signals tend to be Arg-rich. As such, it becomes important that the localization of their WT and AAA mutant proteins be tested. This is also important for their model which proposes a cytoplasmic mechanism.

Response: We thank the reviewer for this important point. We performed cellular fractionation on iSLK and BJAB cells harboring WT, vIRF1-AAA, and vIRF1-REV BAC16 viruses and observed no difference in the subcellular distribution of vIRF1 between the AAA and REV cells across any fraction. The representative blots are now included as Fig. S2d,e, and the text has been updated at line 217.

Comment: 2. Throughout the paper, they over-interpret their AAA mutant phenotypes. Each phenotype is interpreted as being due to RNA-binding. While I think their data identifying and characterizing the binding are strong, it doesn't mean that every phenotype that arises from that mutant is due to that activity. Additional characteristics like localization, folding, or protein-protein interactions could all be altered by that mutation. In some cases (e.g., Fig 5a/b) they have orthogonal approaches, to bolster that interpretation. However, in many cases they do not so they cannot exclude these other models. To be clear, I'm not suggesting that they demonstrate that these phenotypes are specifically due to RNA binding. They simply need to interpret that their observations

appropriately by saying their data are “consistent with” an RNA binding defect, or “depend on the RNA binding motif”. Currently, it seems they interpret every AAA deficiency as “proof” of the importance of RNA binding for that phenotype. Even though I completely agree with the authors that it represents the simplest hypothesis, such statements over-interpret the data.

Response: We thank the reviewer for this well-taken point. We have reworded the text at lines 247, 296, 299, 305, 310, and 331, so that AAA phenotypes are described as “consistent with” an RNA-binding defect or as “dependent on the RNA-binding motif,” rather than as proof of RNA-binding function.

Comment: 3. Similarly, more caution should be taken in interpreting the RNA-dependent interactions in Fig 5. Since vIRF1 binds RNA, it is not surprising that it co-purifies on other abundant RBPs in an RNA-dependent fashion. Whether or not these interactions occur in cells is not demonstrated by these experiments. Again, they do not need to prove this, but they should concede to this limitation.

Response: We thank the reviewer for this insightful point and fully agree. We have now added a closing statement at lines 363–367 acknowledging this limitation. The text now reads “While these interactions were recovered from extracts and the precise cellular context of their assembly remains to be defined, their abolition by RNase and selective loss in the vIRF1-AAA mutant together supports that vIRF1 engages this host RNA–protein network through its RNA-binding activity.”

Reviewer #3 (Remarks to the Author)

Comment: This study applies XRNAX to KSHV infected PEL cells, defining host and viral RNA binding proteomes under latent and lytic conditions and nominating 14 viral RBPs, 7 of which are newly proposed. The follow up centers on vIRF1 (K9): the authors generate a vIRF1AAA mutant that shows reduced RNA binding compared to wildtype. This mutant displays B cell–specific defects in lytic gene expression and virion production that trace to impaired RAS/MEK/ERK activation and an RNA sensitive interaction with G3BP1/2. The work is novel, and the dataset will be a useful resource for the community. The G3BP axis is biologically plausible given literature on RNA driven G3BP conformational switching/condensation and established G3BP signaling roles. Nevertheless, the central mechanistic claim, strict RNA dependence of vIRF1driven MAPK activation, relies largely on a triple alanine substitution that could also perturb protein–protein interfaces. The manuscript would benefit from orthogonal, non mutational tests and a few areas of statistical and validation rigor. Addressing the points below would considerably strengthen the paper’s impact.

Major comments

Comment: 1- the conclusion that vIRF1 activates RAS/MEK/ERK in an RNAdependent manner rests predominantly on the RRR→AAA mutant and RNase sensitive interactions. Since basic patch mutations can also perturb protein–protein interfaces and local electrostatics, it is important to include non mutational evidence of RNA dependence in BJAB: e.g., identify the vIRF1bound RNA(s) (RIPqPCR/eCLIP), RNA decoy/mimic competition that reduces vIRF1–G3BP coIP

Response: We thank the reviewer for this constructive suggestion. To provide non-mutational evidence of RNA-dependence in BJAB cells, we used the MS2 coat protein (MCP) which binds with high affinity to MS2 RNA hairpins, allowing an MCP-fused protein to be recruited onto any transcript bearing those hairpins. BJAB cells were co-transfected with HA-MCP–tagged WT vIRF1 together with either MS2 hairpin-tagged Renilla luciferase (MS2-Rluc) mRNA or an untagged firefly luciferase (Fluc) control. ERK phosphorylation was significantly reduced with MS2-Rluc relative to Fluc (Fig. S5e), and co-immunoprecipitation showed a minor but reproducible reduction in the vIRF1–G3BP1 interaction (Fig. S5f–i). These results support an RNA-dependent mechanism for vIRF1-driven RAS/MEK/ERK activation, consistent with RNA association facilitating the vIRF1–G3BP1 interaction. We would like to note, that our long-term goal is to further define vIRF1 bound RNAs, as well a vIRF1-G3BP co-occupied RNAs, and this will be included in future manuscripts.

Comment: Using a semiquantitative, rule based approach is reasonable only for proteins with complete absence across replicates in one condition (true "on/off" behavior), but for proteins quantified in both conditions, inferential statistics should be applied rather than a hard ratio cutoff. The authors already use limma for the IPMS datasets (p.29–30), so the same moderated test framework (with BH FDR control) should be applied to the XRNAX UV vs No UV comparisons as well. Also in order to reveal the magnitude of RBPome changes during reactivation direct comparison between lytic(+UV) vs. latent(+UV) samples is important.

Response: We thank the reviewer for this suggestion. We applied the same limma moderated t-test framework (with BH FDR control) used for the IP-MS datasets to the XRNAX UV vs. No-UV comparisons; the resulting p-values and adjusted p-values are now included in Supplementary Data 1 and 2. We also performed the direct +UV lytic vs. +UV latent comparison to quantify RBPome changes during reactivation, now provided as Supplementary Data 3, with corresponding text added at line 126.

Comment: 3- The B cell specificity angle is compelling but currently rests only on two lines BJAB vs iSLK that show opposite phenotype. It is important to include more B cell model (a distinct PEL line or primary B cells if feasible).

Response: We thank the reviewer for this suggestion. Primary B cell infection as well as de novo KSHV infection of other B cell lines is notoriously challenging with limited

successes, and indeed we were unable to obtain infected cells for many lines. However, we were successful in establishing latent infections with vIRF1-AAA and vIRF1-REV viruses in EBV-negative Akata cells, a Burkitt's lymphoma–derived B-cell line. Following reactivation with TPA and sodium butyrate (NaB), vIRF1-AAA cells showed marked defects in both viral lytic gene expression (Fig. S2k, l) and ERK phosphorylation (Fig. S4d). These results confirm that the phenotype is a general feature of B cells rather than a BJAB-specific artifact. The text has been updated at lines 242 and 284.

Minor points:

Comment: 1- Statistics reporting inconsistencies & replication clarity: Figure legends state mean ± SD (and elsewhere mean ± SEM) with Student's ttest or oneway ANOVA and the n per experiment aren't uniformly stated.

Response: All bar graphs now present mean ± SD from three biologically independent samples, with significance determined by two-tailed Student's t-test. The figure legends have been updated accordingly.

Comment: 2- "Two aliquots of 500 mg of XRNAX extract were digested with 10 mg trypsin/LysC" line 493. this is supposed to be ug?

Response: Corrected to "10 µg trypsin/LysC."

Comment: 3- The current 32PCLIP validation set is skewed toward previously known or partially characterized viral RBPs, while only a minority of the newly proposed candidates were tested. Please elaborate the criteria used to choose proteins for 32PCLIP.

Response: As noted in the text at line 158, candidates were selected based on our ability to consistently detect their expression and immunoprecipitate them: "We selected 6 candidates, ORF52, vIRF1 (K9), ORF10, ORF11, MIR2 (K5), PAP (ORF59), for verification based on our ability to consistently detect their expression as well as immunoprecipitate them. We note that although ORF10 and ORF52 RNA binding was demonstrated in vitro, their ability to bind RNA directly within cells remained untested, and therefore they were included here."

Comment: Typos: "well-stablished PAMP" → well-established (p.3, line 41–42). "Qualitive analysis" → Qualitative (p.6, line 121–122). "typically rang from 300 to over 2,000" → range (p.6, line 121–123). "bond nucleic acids" → bound nucleic acids (Methods, p.24–25). "laborotaries" → laboratories (Methods, Luciferase assays, p.27, line ~600). "Quantification and statical analysis" → Quantification and statistical analysis (p.30, line 667). "seleced" → selected (Methods, cell line selection, p.25–26). "BAJB" and "BAJBiRTA" → BJAB (multiple instances on p.20–26). "phosphorJNK" → phosphoJNK (Antibody list, p.28, lines 618–621). "p32CLiP" → consistently 32PCLIP (Fig. 3 legend; Fig. 2 panels). "contitutive active ERK" (Figure text overlays) → constitutive.

Response: We thank the reviewer for the careful reading. All identified typos and grammatical errors have been corrected. (Note: we were unable to locate the “constitutive active ERK” figure overlay text; please let us know if it persists in the revised figures.)
